# Therapeutic Potential of Rose Hip-Derived Nanoparticles for Psoriatic Skin Inflammation

**DOI:** 10.1021/acsbiomaterials.5c00826

**Published:** 2025-09-26

**Authors:** Masahiro Hashimoto, Shoko Itakura, Kosuke Kusamori, Katsuhiko Yajima, Shota Mitsuhashi, Shinichiro Hayashi, Hiroaki Todo, Makiya Nishikawa

**Affiliations:** † Faculty of Pharmaceutical Sciences, 378550 Tokyo University of Science, 6-3-1 Niijuku, Katsushika, Tokyo 125-8585, Japan; ‡ Faculty of Pharmacy and Pharmaceutical Sciences, 34771 Josai University, 1-1 Keyakidai, Sakado, Saitama 350-0295, Japan; § Green Flask Laboratory, 1-25-1 Jiyugaoka, Meguro-ku, Tokyo 152-0035, Japan

**Keywords:** plant-derived nanoparticle, extracellular vesicles, skin disease, intradermal injection, psoriasis, rose hip

## Abstract

Psoriasis is a chronic skin disease characterized by hyperproliferation of keratinocytes and excessive inflammation. Plant-derived nanoparticles (pdNPs) are promising agents for treating inflammatory skin diseases. In this study, we examined the characteristics and functions of rose hip-derived nanoparticles (RNPs) rich in various bioactive compounds. RNPs were isolated from rose hips using sucrose ultracentrifugation and characterized using NanoSight and transmission electron microscopy. Cellular uptake by HaCaT human keratinocytes was analyzed using flow cytometry and confocal microscopy. Uptake mechanisms were investigated using siRNA knockdown. Proliferation, apoptosis, and cytokine expression were evaluated in a HaCaT psoriasis model. Antioxidant activity was assessed by measuring reactive oxygen species (ROS) levels in stimulated HaCaT and RAW264.7 mouse macrophage-like cells. The in vivo efficacy was evaluated in a mouse model of psoriasis via intradermal injection of RNPs. The RNPs obtained via ultracentrifugation exhibited a vesicular structure of approximately 100 nm in diameter. They were efficiently taken up by HaCaT cells and inhibited excessive inflammation-induced proliferation. RNPs reduced the mRNA levels of the inflammatory cytokines, interleukin-1β and interferon-γ. Additionally, RNPs were efficiently internalized by mouse macrophage-like RAW264.7 cells, decreasing the intracellular reactive oxygen species levels. The intradermal injection of RNPs effectively suppressed epidermal hyperproliferation and macrophage infiltration in an imiquimod-induced psoriasis mouse model. Collectively, these results suggest that RNPs can be used to treat psoriasis by regulating oxidative stress and inhibiting epidermal hyperproliferation. RNPs, which exerted potent effects on epidermal cells, target key pathological mechanisms such as oxidative stress and immune-driven keratinocyte proliferation. Therefore, they are promising natural therapeutic agents for psoriasis.

## Graphical abstract

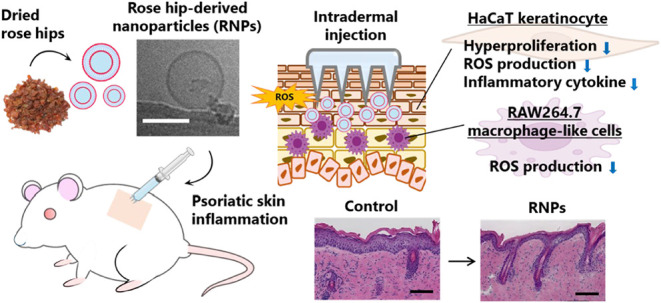

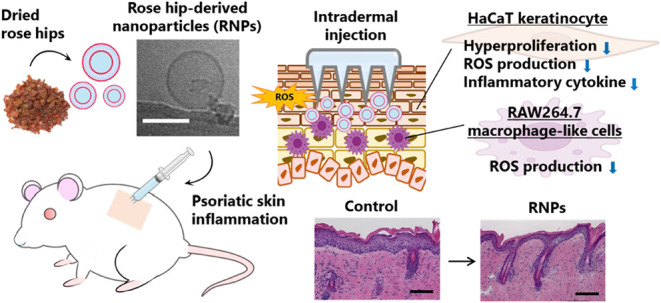

## Introduction

Psoriasis is a chronic inflammatory skin disease characterized by excessive proliferation of keratinocytes and infiltration of immune cells. Although the exact pathogenic mechanisms of psoriasis remain unclear, dysregulated interactions between keratinocytes and immune cells are crucial for its development and progression.^1–3^ Recently, many studies have investigated the role of oxidative stress in the pathogenesis and progression of psoriasis.^4,5^ Defective antioxidant systems and excessive reactive oxygen species (ROS) production in the skin lead to altered cellular transcription factors and signal transduction in psoriasis.^6,7^ ROS production by immune cells, such as macrophages, is crucial for the aberrant proliferation and differentiation of epidermal keratinocytes.^7^ Therefore, in addition to conventional therapies targeting the immune system and suppressing excessive inflammation, strategies focused on ROS inhibition and elimination are useful in treating psoriasis. However, owing to the complex interplay between various factors, existing therapeutic agents, including immunosuppressants, anti-inflammatory agents, and epidermal growth inhibitors, exhibit limited efficacy against psoriasis. Despite their high efficacy, the widespread use of biological agents is limited by their high cost, potential side effects, and high risk of infection. Furthermore, drug delivery to the skin is impeded by the presence of thick plaques, particularly on the palms, soles, and scalp,^8^ underscoring the need for more effective therapeutic strategies that selectively target keratinocytes.

Extracellular vesicles (EVs) are heterogeneous lipid-bilayer nanoparticles secreted by nearly all living cells, including plant cells.^9–11^ These vesicles play a pivotal role in intracellular communication across the kingdom. The general principle of minimal information for studies on EVs (MISEV2023)^12^ apply to plant EVs. Vesicles secreted by plant cells are classified into two primary types: plant EVs obtained from plant apoplasts and cell culture medium, and plant-derived nanoparticles (pdNPs) obtained by crushing and pressing plants.^13,14^ Despite containing a mixture of other membranes, the particle sizes and structures of pdNPs are similar to those of exosomes due to their preparation process. Unlike EVs, which require a labor-intensive preparation method, pdNPs have more applications owing to their simpler preparation method and feasibility for large-scale production. They also show therapeutic potential in cancer and inflammatory diseases, and exert immunomodulatory effects.^15,16^ We previously reported the anticancer activities of corn^17^- and rice bran^18^-derived nanoparticles. These reports highlighted the potential of plant-derived pdNPs as therapeutic agents for various diseases.

Lipid-based nanoparticles such as liposomes,^19^ ethosomes,^20^ transfersomes,^21^ are used to treat skin diseases because of their ability to deliver drugs via multiple skin penetration routes.^22^ Therefore, pdNPs with a lipid bilayer structure are beneficial for treating skin diseases. Plants have various antioxidant and anti-inflammatory components, such as polyphenols, flavonoids, and vitamins; therefore, many studies have focused on identifying safe and effective plants and plant products for psoriasis treatment.^23,24^ In this study, we focused on rose hips, which are the fruits of rose plants traditionally used to treat skin diseases, as a source of pdNPs. Despite the efficacy of rose plant extracts in treating psoriasis,^25^ no studies have explored rose hip-derived pdNPs. Therefore, the potential therapeutic effects of pdNPs in psoriasis warrant further investigation. However, pdNPs are nanosized particles, raising concerns regarding their ability to penetrate rough and thick skin lesions such as plaques. Therefore, in this study, rose hip-derived nanoparticles (RNPs) were characterized, functionally evaluated, and intradermally administered to mice with psoriasis to examine their therapeutic effects and facilitate their application as novel approaches for psoriasis treatment.

## Materials and Methods

### Materials

HaCaT human keratinocytes were purchased from Cell Line Service (Eppelheim, Germany). A375 human malignant melanoma cells were purchased from the Japanese Collection of Research Bioresources Cell Bank (Osaka, Japan). HDFa cells were purchased from Thermo Fisher Scientific (Waltham, MA, USA). RAW264.7 mouse macrophage-like cells were provided by Professor Yoshinobu Takakura (Department of Biopharmaceutics and Drug Metabolism, Graduate School of Pharmaceutical Sciences, Kyoto University, Kyoto, Japan). Dulbecco’s modified Eagle’s medium (DMEM), Roswell Park Memorial Institute medium 1640 (RPMI-1640), trypsin-ethylenediaminetetraacetic acid solution, sucrose, metaphosphoric acid, xylene, and calcitriol were purchased from FUJIFILM Wako Pure Chemical Corporation (Osaka, Japan). dl-homocysteine and 2-methyl-6-phenyl-3,7-dihydroimidazo­[1,2-*a*]­pyrazin-3-one (CLA) were obtained from Tokyo Chemical Industry Co., Ltd. (Tokyo, Japan). Fetal bovine serum was obtained from SERANA (Pessin, Germany). Additionally, 3,3′-dioctadecyloxacarbocyanine perchlorate (DiO) and 1,1’-dioctadecyl-3,3,3′,3′-tetramethylindocarbocyanine perchlorate (DiI) were obtained from PromoCell GmbH (Heidelberg, Germany). LysoTracker Red DND-99, Hoechst 33342, and TRIzol were purchased from Thermo Fisher Scientific. The siRNAs used in this study were synthesized by Fasmac Co., Ltd. (Kanagawa, Japan), and their sequences are listed in Table S1. The negative control siRNA was designed and synthesized by Nippon Gene Co., Ltd. (Tokyo, Japan). Human recombinant oncostatin M, interleukin (IL)-1α, and IL-22 were purchased from PeproTech (Cranbury, NJ, USA). IL-17A and tumor necrosis factor (TNF)-α were obtained from Proteintech Group, Inc. (Rosemont, IL, USA). Xanthine oxidase and Entellan New were purchased from Sigma-Aldrich (St. Louis, MA, USA). The TGX Fast Cast Acrylamide Kit (12%) was purchased from Bio-Rad Laboratories, Inc. (Hercules, CA, USA). The 4% paraformaldehyde phosphate buffer solution and Skim Milk used for the immunoassays were purchased from Nacalai Tesque (Kyoto, Japan). Mayer’s hematoxylin and 1% eosin Y solutions were obtained from Muto Pure Chemicals Co., Ltd. (Tokyo, Japan). Fatty acid standards were purchased from Funakoshi Co., Ltd. (Tokyo, Japan) and prepared using GLC-Reference Standard fatty acid methyl esters (GLC-462). It contained 10:0, 12:0, 12:1, 14:0, 14:1, 16:0, 16:1, 18:0, 18:1n-9, 18:1n-7, 18:2, 18–3n-6, 18:3n-3, 20:0, 20:1n-11, 20:2n-6, 20:3n-6, 20:4n-6, 20:3n-3, 20:5n-3, 22:0, 22:1, 22:2, 22:4, 22:5n-3, 24:0, 22:6n-3, and 24:1n-9 fatty acid methyl esters.

### Animals

Seven-week-old male BALB/c mice were purchased from Sankyo Labo Service Co. (Tokyo, Japan) and maintained under specific-pathogen-free conditions. All animal experiments were approved by the Institutional Animal Experimentation Committee of the Tokyo University of Science (approval number: Y22015) and adhered to the principles and procedures outlined in the National Institutes of Health Guide for the Care and Use of Laboratory Animals and Animal Research: Reporting of In Vivo Experiments.

### RNP Isolation and Characterization

Nanoparticles were isolated from rose hips (Chile) using sucrose cushion ultracentrifugation,^26^ with some modifications. Briefly, 100 g of dried rose hips was crushed in 300 mL of phosphate-buffered saline (PBS) and centrifuged first at 3000 × *g* for 10 min and then at 10 000 × *g* for 60 min. The supernatant was passed through a 0.45-μm filter. For sucrose cushion isolation, 15 mL was layered over 1.5 mL of 30% sucrose in PBS. The mixture was ultracentrifuged at 141 000 × *g* for 90 min at 4 °C using CP80WX with swing rotor P40ST (Eppendorf Himac Technologies Co., Ltd., Ibaraki, Japan). The sucrose layer was washed using ultracentrifugation, resuspended in PBS, and centrifuged at 21 600 × *g* for 30 min. RNPs were collected from the supernatant, and their size distribution was measured using a NanoSight NS300 (Malvern Panalytical Ltd., Malvern, UK). Polydispersity index and zeta potential were measured using ELSZ-2000 (Otsuka Electronics Co., Ltd., Osaka, Japan). The protein concentrations in the RNPs were determined using a BCA protein assay kit (Nacalai Tesque). MicroRNAs (miRNAs) in the RNPs were collected using a microRNA Extractor Kit for Purified EV (FUJIFILM Wako Pure Chemical Corporation), and their concentrations were determined using a Qubit microRNA Assay Kit (Thermo Fisher Scientific).

### Transmission Electron Microscopy (TEM) and Cryogenic (cryo)-TEM

The RNP morphology was observed using TEM and cryo-TEM. A drop of RNPs was deposited onto a carbon-coated copper grid for 1 min and negatively stained with 1% sodium phosphotungstate for 1 s for TEM. The sample was allowed to dry at room temperature (approximately 22–25 °C) prior to observation using H-7650 TEM (Hitachi High-Tech Co., Ltd., Tokyo, Japan) at 100 kV. RNPs (1 μL of 5 mM lipid concentration) were applied to hydrophilized copper grids (200 mesh; JEOL Ltd., Akishima, Tokyo, Japan) and blotted for cryo-TEM. Samples were frozen using a rapid freezing system (EM-CPC; Leica Microsystems, Tokyo, Japan) and observed using an EM-3100FEF cryo-TEM (JEOL Ltd.) at an acceleration voltage of 300 kV.

### Evaluation of RNP Cellular Uptake

The RNPs were labeled with DiO (excitation, 484 nm; emission, 501 nm). A 1 μL solution of 1 mM DiO-ethanol was added to the RNP suspension containing 50 μg of protein, and the mixture was incubated for 30 min. Subsequently, the mixture was subjected to ultracentrifugation to remove unbound dye. HaCaT, A375, and HDFa cells (4 × 10^4^ cells/well) were seeded in 24-well plates and treated with 5, 10, and 50 μg/mL DiO-labeled RNPs for 3–24 h at 37 °C. The cellular uptake of RNPs was analyzed using a flow cytometer (FACSLyric; BD Biosciences, Tokyo, Japan). The mean fluorescence intensity ratio (MFIR) was calculated by dividing the fluorescence intensity of the sample by that of untreated cells. Two separate experiments were performed to assess the cellular uptake pathway, one using chemical inhibitors and the other using siRNA-mediated gene knockdown. The cells were treated with 0.4 M sucrose (clathrin-mediated endocytosis inhibitor) or 5 μM rottlerin (macropinocytosis inhibitor) in the inhibitor experiment. For gene knockdown experiments, siRNAs targeting DNM2 and Rac1 were transfected into HaCaT cells using Lipofectamine RNAiMax (Thermo Fisher Scientific) and incubated for 48 h. The culture medium was replaced with fresh DMEM and the cells were reseeded, followed by treatment with DiO-RNPs. Fluorescein isothiocyanate transferrin (20 μg/mL) and FD70 (500 μg/mL) were used as markers of clathrin-mediated endocytosis and macropinocytosis, respectively.

### Evaluation of RNP Intracellular Trafficking

Cells (1.5 × 10^5^ cells) were seeded in 35 mm glass dishes and treated with 50 μg/mL of DiO-labeled RNPs at 37 °C for 6 h. Endosomes/lysosomes and nuclei were stained with 1 μM of LysoTracker Red DND-99 (excitation: 577 nm; emission: 590 nm) and 10 μg/mL of Hoechst 33342 (excitation: 352 nm; emission: 461 nm), respectively. Images were obtained using a confocal laser-scanning microscope (CLSM) SP8 LIGHTNING (Leica, Wetzlar, Germany) equipped with a 63× water immersion objective lens (HC PL APO 63×/1.20 W CORR CS2).

### Antioxidant Component Analysis via High-Performance Liquid Chromatography

Ascorbic acid, β-carotene, and gallic acid levels in RNPs were determined using a high-performance liquid chromatography system (Prominence; Shimadzu, Kyoto, Japan) with a UV detector (SPD-20A; Shimadzu). For ascorbic acid, the RNPs were treated with 2% metaphosphoric acid, and total ascorbic acid was measured after reduction with 0.1% homocysteine and 10% sodium phosphate. Separation was performed using the COSMOSIL HILIC column (4.6 × 150 mm, 5 μm) at 30 °C with a 1 mL/min mobile phase (acetonitrile:ammonium acetate (50 mM); 50:50 (v/v)) and detected at 254 nm. For β-carotene, RNPs were extracted using methanol and chloroform, and centrifuged at 1500 × *g* for 10 min. The lower phase was injected into the COSMOSIL 5C18-MS-II column (4.6 × 150 mm). Separation was performed at 30 °C with a 1 mL/min mobile phase (chloroform:methanol; 30:70 (v/v)) and detected at 450 nm. For gallic acid, RNPs were extracted with methanol and centrifuged at 10 000 × *g* for 10 min. The resulting upper layer was injected into a COSMOSIL 5C18-MS-II column. Separation was performed at 30 °C with a 1 mL/min mobile phase (methanol:phosphate buffer (20 mM; pH 2.5); 10:90 (v/v)), and detection was performed at 272 nm.

### Fatty Acid Analysis via Gas Chromatography

Dried rose hips (100 mg) and RNPs (100 μL) were prepared for fatty acid analysis. Fatty acid profiles were determined using gas chromatography as previously described.^27^ BHT/methanol (0.005%) and tricosanoic acid as internal standards were added to each sample and stored at −30 °C. Samples were heated at 98 °C for 1 h after adding acetyl chloride and shaken for 3 min after the sequential addition of 0.5 M sodium hydroxide/10% sodium chloride and octane. The samples were centrifuged at 1000 × *g* for 10 min at 20 °C, and the top layer was collected. The RNPs were nitrogen-dried and dissolved in 50 μL of octane. The total fatty acid composition was measured using gas chromatography (GC-2014; Shimadzu) with a flame ionization detector and an automatic sampler (AOC-20i; Shimadzu). Analysis used a capillary column (DB-WAX 30 m × 0.53 mm × I.D 3 μm), split injection with a 10:1 ratio, and nitrogen as carrier gas. The column temperature was held at 100 °C for 4 min, increased to 180 °C at 20 °C/min and held for 3 min, then ramped to 200 °C at 4 °C/min, followed by an increase to 230 °C at 1 °C/min and held for 5 min. The run time for each sample was 49 min. The fatty acids were identified based on the retention times of the standards and quantified as the ratio of each peak area to that of the internal standard. The total fatty acid content was then calculated.

### Phospholipid Analysis via Liquid Chromatography-Tandem Mass Spectrometry

The lipid components of the RNPs were analyzed using the liquid chromatography-tandem mass spectrometry package for phospholipid profiling (Shimadzu, Kyoto, Japan), according to the manufacturer’s instructions. Briefly, the RNPs (1 × 10^11^) were added to a methanol solution containing 0.1% formic acid (Tokyo Chemical Industry Co., Ltd.) for mass spectral analysis. Subsequently, 5 μL of the sample solution was injected and analyzed using LCMS-8050 (Shimadzu), as previously described.^28^

### ROS-Scavenging Ability of RNPs

Next, 10 μL of RNPs (10, 50, and 100 μg/mL) or ascorbic acid (50 μM) was combined with 90 μL of hydrogen peroxide (H_2_O_2_; 10 μM) in a tube. The mixture was incubated for 1 h at room temperature. H_2_O_2_ levels were determined using a Red H_2_O_2_ Assay Kit (Enzo Life Sciences, NY, USA). Hydroxyl radical levels were determined using an antioxidant capacity assay kit (SAKULABSCIENCE, Yokohama, Japan). In a 96-well plate, 10 μL of RNPs (10, 50, and 100 μg/mL) or ascorbic acid (50 μM) was mixed with 1 μL of xanthine oxidase (0.12 U/mg) and 94 μL of CLA diluted 1:20. After the addition of 5 μL xanthine, luminescence was measured using a microplate reader (GloMax; Promega, WI, USA). Intracellular ROS-scavenging activity was evaluated using the ROS Assay Kit-Highly Sensitive DCFH-DA (Dojindo, Kumamoto, Japan). HaCaT and RAW264.7 were treated with RNPs (10, 50, 100 μg/mL) for 3 h, followed by incubation with DCFH-DA at 37 °C for 30 min. Fluorescence intensity was measured using a flow cytometer (FACSLyric), and images were acquired using a BZ-X800 microscope (KEYENCE, Osaka, Japan). Furthermore, intracellular levels of superoxide anion radicals were evaluated using the ROS Detection Cell-Based Assay Kit (Cayman Chemical, Ann Arbor, MI, USA) and dihydroethidium (DHE) staining. HaCaT cells were treated with RNPs (50 μg/mL) and N-acetylcysteine (NAC, 5 mmol/L), washed, DHE-stained, and stimulated with antimycin A (150 μM). After 1 h, fluorescence was observed using a microscope (KEYENCE BZ-X800) and measured with a microplate reader (GloMax) at an excitation wavelength of 520 nm and an emission range of 580–640 nm.

### Cell Growth Assay

HaCaT, HDFa, and A375 cells (4 × 10^3^ cells/well) were seeded into 96-well plates. Following overnight incubation at 37 °C, the cells were treated with RNPs (0.1–100 μg/mL) for 48 h. HaCaT cells were treated with 10 nM active vitamin D_3_ (calcitriol) as the positive control. The cell number was determined using a cell counting kit-8 (Dojindo Laboratories). In addition, HaCaT cells were stimulated with 2.5 ng/mL of five cytokines, i.e., oncostatin M, IL-17A, TNF-α, IL-1α, and IL-22 (M5), to establish psoriasis model cells.^29^

### Apoptosis and Cell Cycle Assays

HaCaT cells (4 × 10^4^ cells/well) were seeded in 24-well plates and treated with 50 μg/mL of RNPs and 2.5 ng/mL of M5 for 48 h at 37 °C. Dead cells were detected using an Annexin V-633 Apoptosis Detection Kit (Nacalai Tesque). The nuclei were stained with Cell Cycle Assay Solution Blue (Dojindo Laboratories) for cell cycle analysis. Finally, the cells were analyzed using a BD FACSLyric flow cytometer with FlowJo software version 8.7 (Becton, Dickinson and Biosciences, Franklin Lakes, NJ, USA).

### Western Blotting

HaCaT cells were washed with phosphate-buffered saline (PBS) and lysed using radioimmunoprecipitation assay buffer. The resulting precipitate was separated from the soluble fraction using centrifugation at 21 600 × *g* for 20 min. The resulting protein extracts (12 μg) were subjected to 12% sodium dodecyl sulfate-polyacrylamide gel electrophoresis (SDS-PAGE; TGX FastCast Acrylamide Kit) and fractionated at 150 V. Subsequently, the gels were transferred onto polyvinylidene fluoride membranes (Immobilon-P membranes; Merck Millipore, Darmstadt, Germany) at 100 V for 90 min. After blocking with blocking buffer (5% skim milk), the membranes were incubated with primary antibodies against phospho-Akt (#4060; Cell Signaling Technology, Inc., Danvers, MA, USA), Akt (#4060; Cell Signaling Technology, Inc.), cyclin B1 (67686–1-Ig; Proteintech Group, Inc.), cyclin D1 (ZRB1313; Sigma-Aldrich), Nrf2 (#12721, Cell Signaling Technology), p-Nrf2 (#F1552, Selleck Chemicals LLC), OH-1 (#82551, Cell Signaling Technology, Inc.) and glyceraldehyde-3-phosphate dehydrogenase (016–25523; FUJIFILM Wako Pure Chemical Corporation) in a blocking buffer at 4 °C overnight. After washing with Tris-buffered saline containing Tween, the membranes were incubated with horseradish peroxidase (HRP)-labeled antirabbit and antimouse IgG secondary antibodies (#7074 and #7076, respectively; Cell Signaling Technology, Inc.) in blocking buffer at room temperature for 1 h. The membranes were washed three times with Tris-buffered saline containing Tween and incubated with Immobilon Western Chemiluminescent HRP substrate (Merck Millipore). The images were captured using an iBright FL1000 microscope (Thermo Fisher Scientific).

### Evaluation of mRNA Expression via Quantitative Reverse Transcription-Polymerase Chain Reaction (RT-qPCR)

HaCaT cells (4 × 10^4^ cells/well) in 24-well plates were treated with 5, 10, and 50 μg/mL of RNPs and 2.5 ng/mL of M5 for 24 h at 37 °C. Total RNA was extracted from samples using the FastGene RNA Basic Kit (NIPPON Genetics Co., Ltd., Tokyo, Japan) and reverse-transcribed into cDNA using the ReverTra Ace qPCR RT Master Mix with gDNA Remover (FSQ-301; TOYOBO, Osaka, Japan). RT-qPCR was performed using the THUNDERBIRD SYBR qPCR Mix (QPS-201; TOYOBO) following the manufacturer’s instructions on a CFX Connect Real-Time System (Bio-Rad Laboratories, Inc.). All primers used to amplify the target genes are listed in Table S2. The 2^–ΔΔCt^ method was used to calculate the fold changes in gene expression, which was normalized to that of glyceraldehyde-3-phosphate dehydrogenase. Full-thickness skin samples (1 cm × 1 cm) were excised from the RNP application site within the IMQ-treated area (1.5 cm × 1.5 cm) for in vivo skin analysis. The sampling area was standardized across animals to ensure consistency. Tissues were immediately homogenized in TRIzol reagent for total RNA extraction and mRNA expression levels of keratin (*KRT*)-6, *TNF-α*, and *IL-1β* were analyzed as described above.

### In Vivo RNP Treatment of Psoriasis Model Mice

We used a widely used and standardized protocol, as reported in previous studies,^30^ to induce psoriasis-like skin inflammation and establish a mouse model of psoriasis. Briefly, the mice received daily topical application of imiquimod (IMQ) cream (BESELNA Cream 5%; Mochida Pharmaceutical Co., Ltd., Tokyo, Japan) at 20 mg/cm^2^ on a shaved back every morning for 7 consecutive days. RNPs (100 μg/mL) were administered via 50 μL intradermal injection using a hollow microneedle (hMN) (MicronJet; NanoPass Technologies Ltd., Nes Ziona, Israel) 8 h after IMQ application, to enable sufficient time for IMQ to permeate into the skin and avoid interference from residual cream. Changes in topical application, intravenous injection, and subcutaneous injection were compared. In brief, RNPs (100 μg/mL in PBS, 50 μL) were administered once daily via intravenous (IV) injection into the tail vein using a 29-gauge needle, subcutaneous (SC) injection under the IMQ-treated dorsal skin using a 26-gauge needle, or topical application by spreading the same volume directly onto the IMQ-treated skin surface. PBS was used as the vehicle control.

### Skin Distribution of RNPs

DiI-labeled RNPs were administered to the dorsal skin of IMQ-treated mice by topical application or intradermal (ID) injection using hMN (MicronJet). For topical application, 50 μL of DiI-RNPs (100 μg/mL) was applied to the skin surface, and imaging was performed 1 h later after confirming that the applied solution had visibly disappeared from the skin. For ID injection, 50 μL of DiI-RNPs was injected and imaged immediately. After administration, the skin areas were harvested, embedded in SCEM compound (Section-Lab Co. Ltd.), and 10 μm frozen sections at the injection site were prepared using a cryostat (Leica). Skin sections were visualized using a CLSM SP8 LIGHTNING (Leica, Wetzlar, Germany) with a 10× objective lens (HC PL APO 10x/0.40).

### Skin Tissue Staining

After in vivo treatment with RNPs, 10 μm frozen skin sections at the injection site were prepared using a cryostat (Leica). After hematoxylin and eosin staining, the sections were fixed in 4% paraformaldehyde for 10 min, stained with hematoxylin for 1 min, and rinsed under running tap water for 10 min, with water changes as necessary, to allow for color development. The sections were stained with eosin for 1 min and washed with distilled water. Sections were sequentially dehydrated with ethanol, cleared with xylene, and mounted with Entellan. Subsequently, Ki-67, F4/80, Ly6*G*/6C, and HO-1 expression levels were determined using fluorescence immunostaining. Briefly, the sections were fixed, blocked with 1% bovine serum albumin for 30 min, and incubated with primary antibodies against Ki-67 (14–5698–82; Thermo Fisher Scientific), F4/80 (14–4801–82; Thermo Fisher Scientific), Ly6*G*/6C (14–5931–82; Thermo Fisher Scientific), and HO-1 (82551; Cell Signaling Technology) at 4 °C overnight. After washing, sections were incubated with Alexa488-conjugated secondary antibodies (A11006; Thermo Fisher Scientific) or Alexa647-conjugate secondary antibodies (A31573; Thermo Fisher Scientific) and observed under a BZ-X800 microscope (KEYENCE, Osaka, Japan).

### Statistical Analyses

Statistical analyses were conducted via analysis of variance, followed by Dunnett’s test, using JMP Pro version 17.2.0 software (SAS Institute, Cary, NC, USA). The following *p*-values were considered statistically significant: *p < 0.05, **p < 0.01, and ***p < 0.001.

## Results

### RNP Collection and Characterization

Nanoparticles were isolated and purified from dried rose hips via ultracentrifugation using a sucrose cushion, as previously described.^25^ Figure 1a,b shows the morphology of the purified nanoparticles as determined using TEM and cryo-TEM, respectively. Nanoparticles with a size of approximately 100–200 nm, along with a lipid bilayer and a single lamellar layer, were identified as RNPs. Figure 1c shows the particle size distribution of the RNPs, indicating that the RNPs exhibit a specific width, which is consistent with the image in Figure 1a. The RNP characteristics are presented in Table 1. The RNPs were negatively charged and had an average size of approximately 170 nm. The recovery yield of the RNPs was approximately 7 × 10^9^ per gram of dried rose hips. The RNPs were found to contain proteins and microRNAs (miRNAs). The major phospholipid components of the RNPs were identified as phosphatidylcholine (PC), phosphatidylethanolamine (PE), and phosphatidylserine (PS). PE was hardly detected in the supernatant after ultracentrifugation but was concentrated in the RNPs (Figure S1). Subsequently, the bioactive components of RNPs, including antioxidants and fatty acids, were quantitatively evaluated. Analysis of the composition of RNPs, the primary antioxidants in rose hips, revealed similar amounts of β-carotene and gallic acid; however, no ascorbic acid was detected (Figure 1d). The fatty acid composition of RNPs differed from that of rose hips; the levels of C18:2n-6 and C18:3n-3, which were high in rose hips, decreased, whereas those of C16:0 and C18:0 increased (Figure 1e).

**Figure 1:**
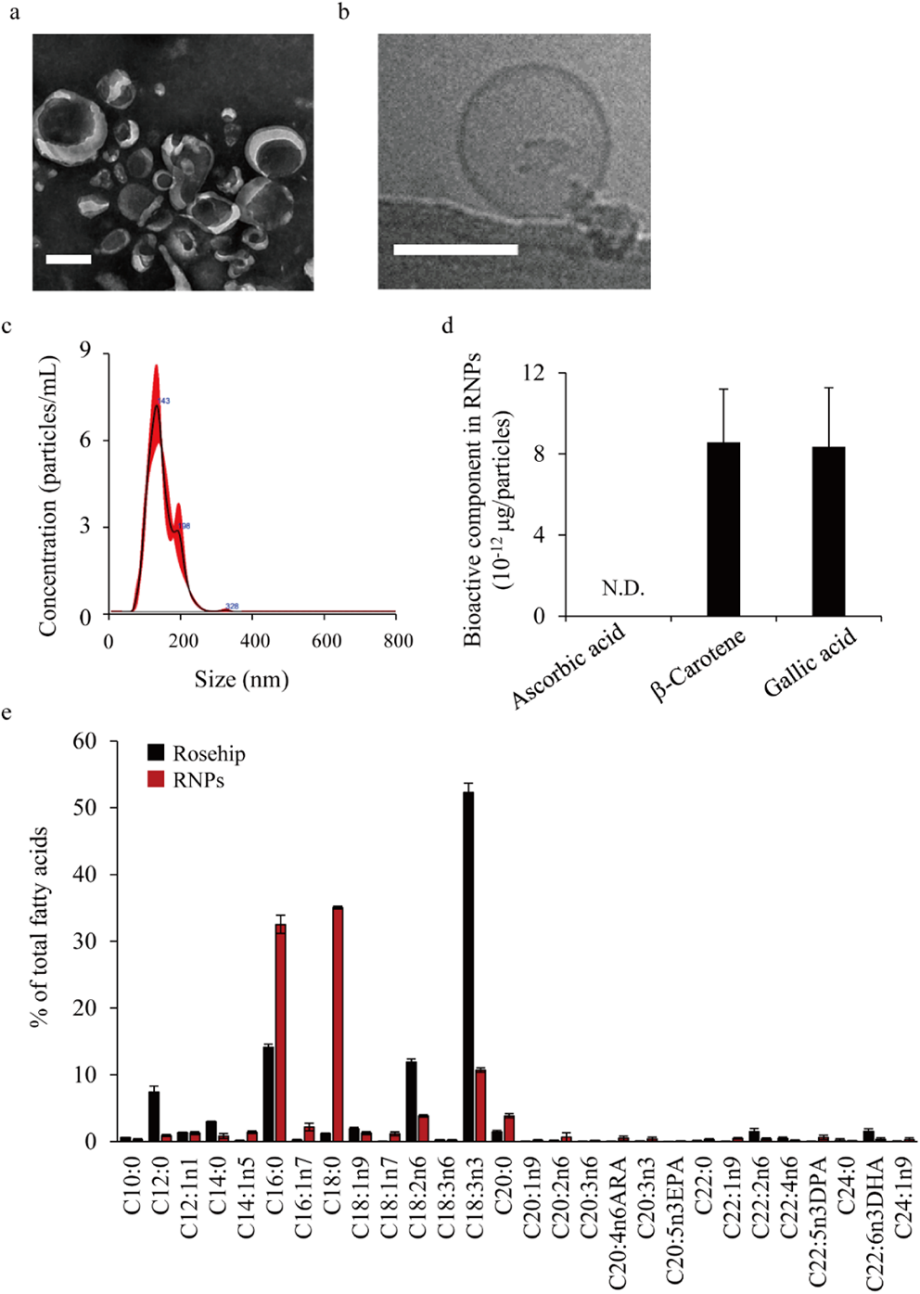
Preparation and characterization of the rose hip-derived nanoparticles (RNPs). (a) A large area of RNPs in the transmission electron microscopy (TEM) image. Scale bar, 100 nm. (b) Enlarged image of RNPs in the cryogenic (cryo)-TEM image. Scale bar, 100 nm. (c) Size distribution of RNPs determined via nanotracking. (d) Ascorbic acid, β-catenin, and gallic acid levels in RNPs determined using high-performance liquid chromatography (HPLC). Data are represented as the mean ± standard deviation (SD; *n* = 3). N.D., not detected. (e) Fatty acid levels in rose hips and RNPs were determined via gas chromatography (GC).

**Table 1:** Characterization of the Rose Hip-Derived Nanoparticles (RNPs)^a^

Average size (nm)	Zeta potential (mV)	Particle number (particles/g)	Protein amount (ng/particle)	miRNA amount (pg/particles)
174 ± 45.0	–13.8 ± 2.88	6.29 × 10^9^	2.02 × 10^–6^	9.2 × 10^–9^

a Data are represented as the mean ± SD (*n* = 3).

### RNP Uptake by HaCaT Cells

Skin-related cell lines were treated with DiO-labeled RNPs for 6 h, and their uptake was assessed using flow cytometry. Figure 2a,b shows the calculated mean fluorescence intensity ratios of DiO-positive cells. The uptake of RNPs by HaCaT cells was more than 3-fold higher than that by A375 and HDFa cells (Figure 2a). RNPs did not significantly change the A375 and HDFa cell numbers at the tested concentrations (0.1–100 μg/mL) but decreased the HaCaT cell number at concentrations above 10 μg/mL (Figure S2). Notably, RNPs increased the cellular uptake in a concentration- and time-dependent manner (Figure 2a,b). Subsequently, the intracellular localization of the RNPs was observed using CLSM. Figure 2c shows a representative CLSM image of the HaCaT cells. Extensive colocalization was observed between green dots derived from DiO-labeled RNPs and red dots, indicating endosomes/lysosomes in the cells. Furthermore, the cellular uptake pathway of RNPs by HaCaT cells was assessed after treatment under low-temperature conditions and the knockdown of endocytosis-related proteins. Figure 2d shows the almost complete reduction in RNP uptake by HaCaT cells at 4 °C compared to that at 37 °C, indicating that it is involved in an energy-dependent pathway. Additionally, a reduction in the levels of *DNM2*, which plays a critical role in clathrin-mediated endocytosis of vesicle fission, and *Rac1*, which plays a critical role in regulating macropinosome formation, suppressed RNP uptake by HaCaT cells (Figure 2e,f). The knockdown efficiencies of DNM2 and Rac1 were confirmed by Western blotting (Figure S3a). Moreover, sucrose and rottlerin, clathrin-mediated endocytosis, and macropinocytosis inhibitors significantly suppressed the cellular uptake of RNPs (Figure S3b).

**Figure 2:**
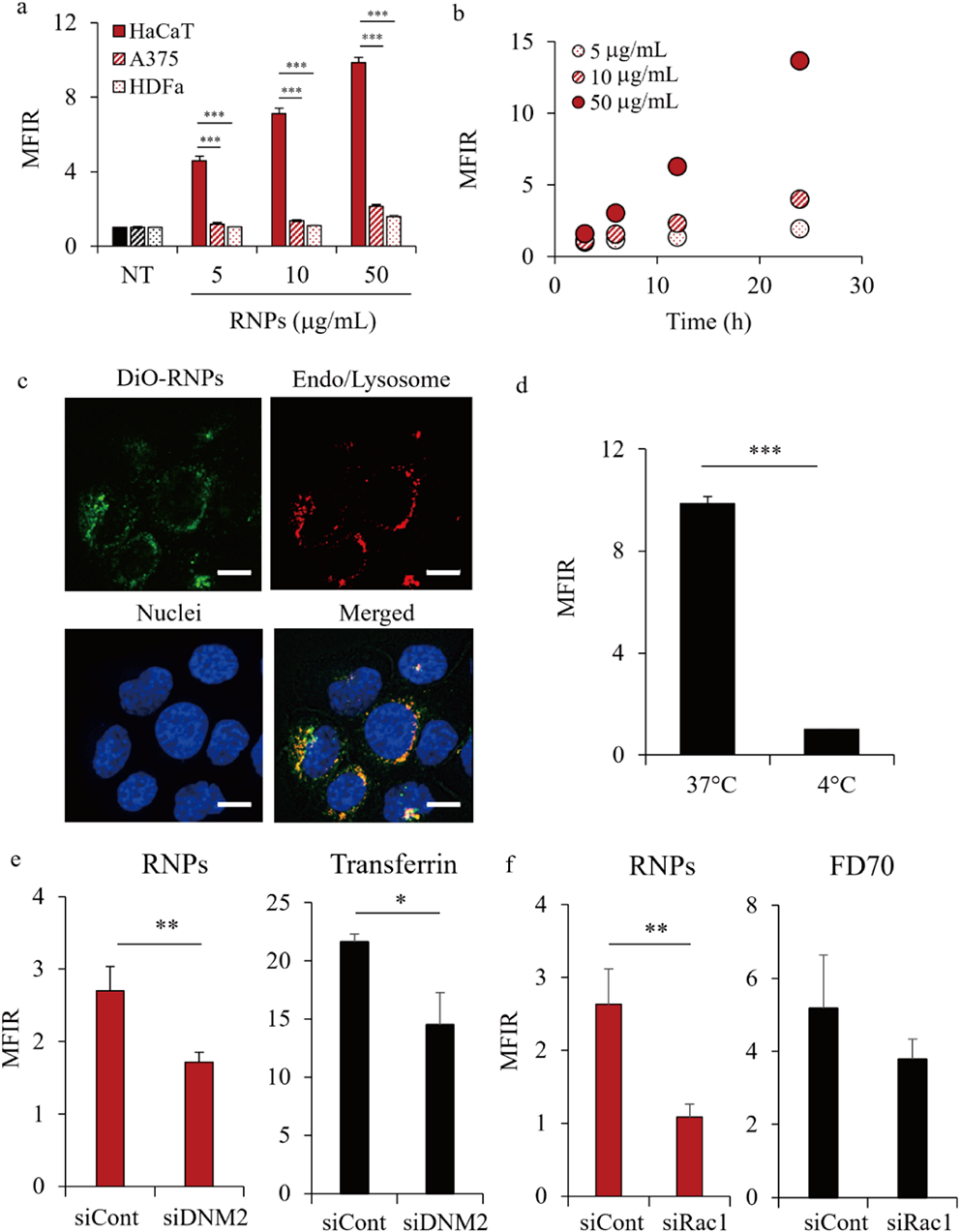
Intracellular uptake of RNPs by HaCaT cells. (a) Cellular uptake of RNPs by HaCaT, A375, and HDFa cells. The cells were incubated with 3,3′-dioctadecyloxacarbocyanine perchlorate (DiO)-labeled RNPs (DiO-RNPs) at 37 °C for 6 h. Fluorescence intensity of individual cells was determined using flow cytometry, and cellular uptake was represented as the mean fluorescence intensity ratio (MFIR). Data are represented as the mean ± SD (*n* = 3). ***p < 0.001. (b) Time-dependent cellular uptake of DiO-RNPs in HaCaT cells using flow cytometry. Cells were incubated with DiO-RNPs at 37 °C for 3, 6, 12, and 24 h. Data are represented as the mean ± SD (*n* = 3). (c) Intracellular localization of RNPs in HaCaT cells. The cells were seeded in a glass-bottom dish, incubated with DiO-RNPs (green) at 37 °C for 6 h, stained with Hoechst 33342 (blue) and LysoTracker Red (red), and observed using confocal laser-scanning microscopy (CLSM). Scale bars, 10 μm. Cellular uptake of RNPs by HaCaT cells at 4 °C (d) under dynamin 2 (*DNM2*) knockdown (e) and Ras-related C3 botulinum toxin substrate 1 (*Rac1*) knockdown (f) was similarly evaluated using flow cytometry. Transferrin and FD70 were used as markers for internalization via clathrin-mediated endocytosis and macropinocytosis, respectively. Data are represented as the mean ± SD (*n* = 3). *p < 0.05 and **p < 0.01.

### Antioxidant Activity of RNPs

Figure 3 shows the antioxidant activity of the RNPs. The evaluation of their ROS elimination capacity revealed that the RNPs scavenged H_2_O_2_ (Figure 3a) and hydroxyl radicals (Figure 3b), but not superoxide anion radicals (Figure 3c), in a concentration-dependent manner. Figure 3d,e shows the scavenging activity of RNPs against ROS produced by HaCaT cells, as determined via fluorescence microscopy and flow cytometry using an ROS-detecting fluorescent probe (2’,7’-dichlorodihydrofluorescein diacetate; DCFH-DA). H_2_O_2_ stimulation increased the green signal of the probe in HaCaT cells; however, this effect was attenuated in HaCaT cells preincubated with RNPs for 3 h (Figure 3d). Flow cytometry revealed an approximately one-third reduction in the fluorescence intensity after RNP treatment (Figure 3e). In addition to DCFH-DA staining, intracellular superoxide anion radical levels were evaluated using DHE staining. Fluorescence microscopy and quantitative analysis revealed that antimycin A increased superoxide anion radical levels, which were not significantly affected by treatment with RNPs (Figure 3f,g). The expression levels of Nrf2-related proteins were analyzed to further investigate the involvement of endogenous antioxidant pathways in the ROS-scavenging activity of RNPs. Western blot analysis showed that H_2_O_2_ increased the phosphorylation of Nrf2 and expression of HO-1, whereas pretreatment with RNPs suppressed this upregulation (Figure 3h,i).

**Figure 3:**
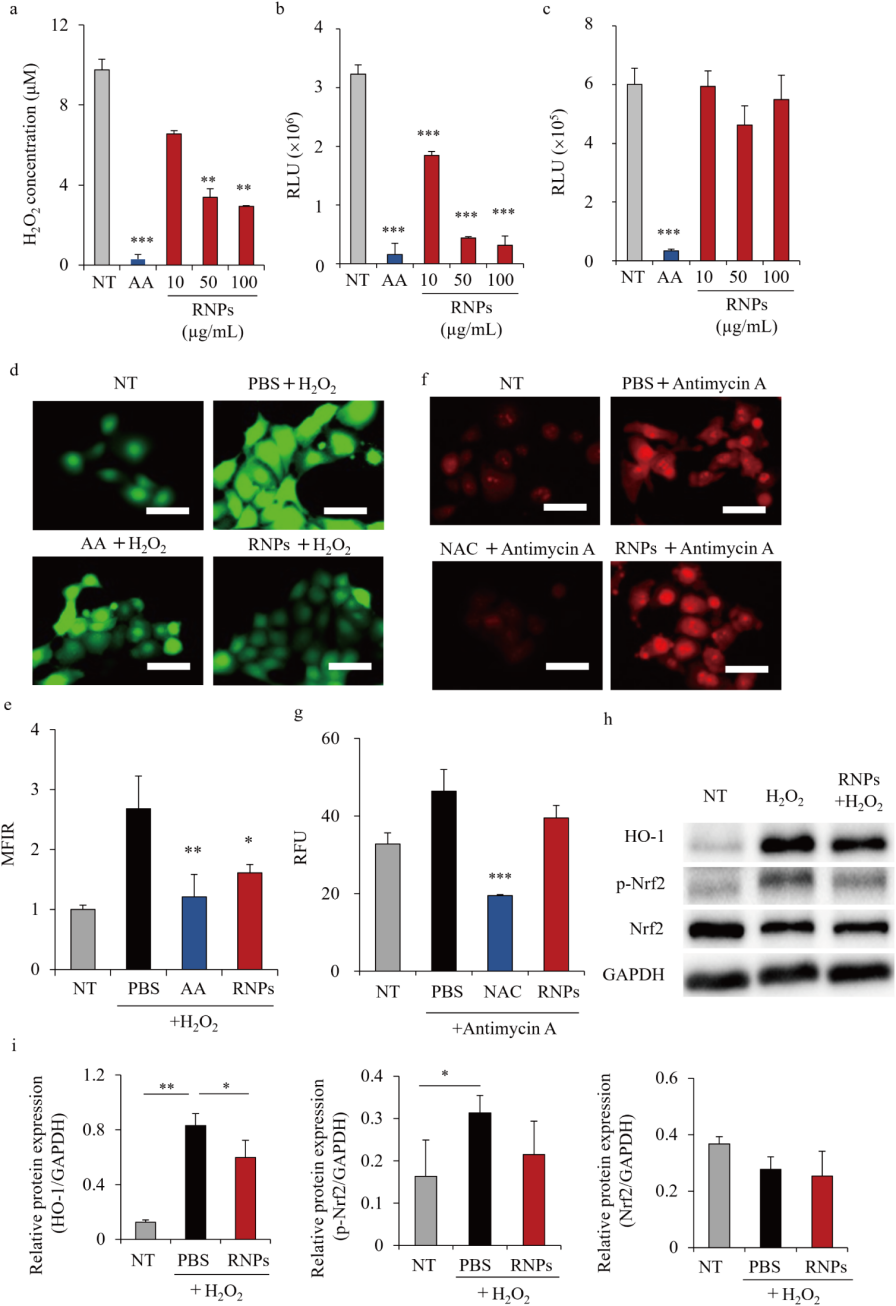
Antioxidant activity of RNPs. Analysis of the reactive oxygen species (ROS)-scavenging activity of RNPs. Hydrogen peroxide (H_2_O_2_) (a), hydroxyl radical (b), and superoxide anion radical (c) levels were measured after reaction with 10, 50, and 100 μg/mL of RNPs. Ascorbic acid (AA) was used as an antioxidant control. Data are represented as the mean ± SD (*n* = 3). **p < 0.01 and ***p < 0.001 vs NT. HaCaT cells were first incubated with 50 μg/mL of RNPs at 37 °C for 3 h and then with 700 μM H_2_O_2_ for 3 h, stained with the 2’,7’-dichlorodihydrofluorescein diacetate (DCFH-DA) dye, and observed via fluorescence microscopy (d, Scale bars, 50 μm) and flow cytometry (e). Data are represented as the mean ± SD (*n* = 3). *p < 0.05 and **p < 0.01. NT, no treatment. To measure superoxide anion radicals, cells were stimulated with antimycin A (150 μM). *N*-acetylcysteine (NAC) was used as antioxidant control acting as scavengers of superoxide anion radicals. Superoxide anion radicals were stained with dihydroethidium (DHE) for 1 h and observed using fluorescence microscopy (f, Scale bars, 50 μm) and detected using a fluorescent microplate reader (g). Data are represented as the mean ± SD (*n* = 3). ***p < 0.001, vs PBS. NT, no treatment. (h) Western blotting analysis of HO-1, phosphorylated Nrf2 (p-Nrf2), and total Nrf2 in HaCaT cells. Cells were pretreated with RNPs (50 μg/mL) for 3 h, followed by stimulation with H_2_O_2_ (700 μM) for 24 h. (i) Relative HO-1, Nrf2, and p-Nrf2 expression levels, normalized to that of GAPDH levels, were analyzed using the ImageJ software. Data are represented as the mean ± SD (*n* = 3). *p < 0.05, **p < 0.01.

### Effect of RNPs on Psoriasis-Like Cell Growth

A cocktail of five cytokines, M5 (IL-17A, IL-22, oncostatin M, IL-1α, and TNF-α), is widely used to stimulate HaCaT cells in psoriasis-like keratinocyte models.^31,32^ Figure 4a–c shows the effects of RNPs on M5-stimulated HaCaT cell proliferation. M5 stimulation significantly promoted HaCaT cell proliferation, which was inhibited by the RNPs in a concentration-dependent manner (Figure 4a). The overgrowth of M5-stimulated HaCaT cells and suppression by RNPs were also observed in the cell images (Figure 4b). Notably, addition of PS-liposomes did not inhibit M5-stimulated HaCaT cell proliferation (Figure S4a). As a positive control, active vitamin D_3_ (calcitriol), a clinically used antipsoriasis agent, significantly reduced HaCaT cell proliferation (Figure S4b). Moreover, HaCaT cells pretreated with RNPs exhibited suppressed M5-induced hyperproliferation, similar to that observed after continuous exposure (Figure S4c). We also measured the expression of *KRT6*, a gene involved in psoriatic keratinocyte overgrowth. Consistent with the cell count results (Figure 4a,b), the addition of RNPs significantly reduced *KRT6* mRNA levels in a concentration-dependent manner (Figure 4c). Figure 4d shows the expression levels of inflammatory cytokines in the psoriasis-like cells. Levels of the inflammatory cytokines, interferon-γ and IL-1β, were increased by M5 stimulation; however, RNPs reversed this effect in a concentration-dependent manner.

**Figure 4:**
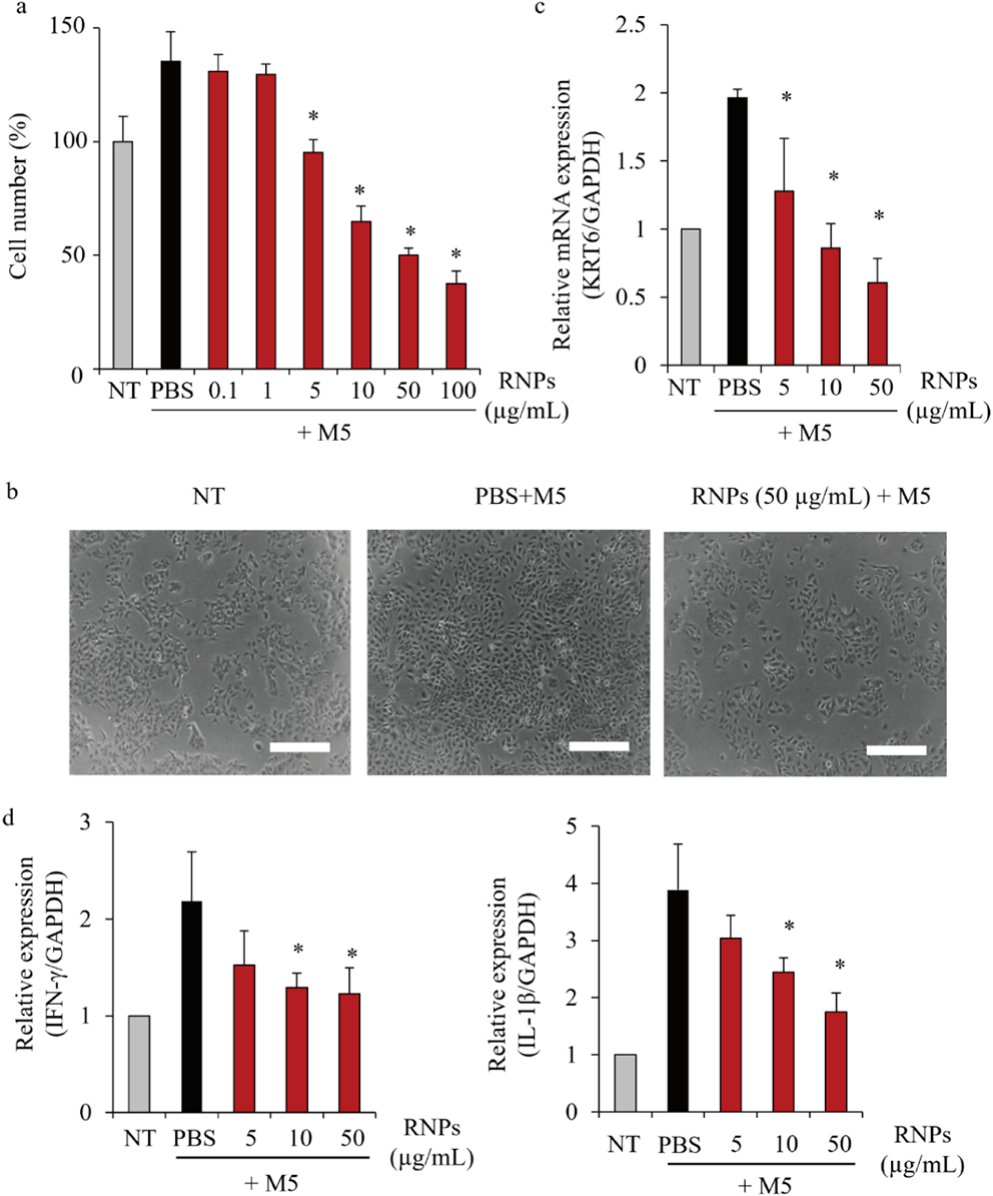
Effect of RNPs on cell growth. (a) Effect of RNPs on M5-stimulated HaCaT cell proliferation. The cells were incubated with RNPs at 37 °C for 48 h, and cell number was measured using the cell counting kit (CCK)-8 assay. Data are represented as the mean ± SD (*n* = 4). *p < 0.05 vs phosphate-buffered saline (PBS) + M5. (b) Representative images of HaCaT (NT) and psoriasis-like HaCaT cells 48 h after incubation with PBS (PBS + M5) or RNPs (RNPs + M5). PBS was used as a vehicle control. Scale bars, 200 μm. Keratin 6 (*KRT6*) (c) and interferon (*IFN*)-γ and interleukin (*IL*)-*1β* (d) mRNA levels in M5-stimulated HaCaT cells incubated with RNPs at 37 °C for 24 h were determined using quantitative reverse transcription-polymerase chain reaction (RT-qPCR). Glyceraldehyde-3-phosphate dehydrogenase (GAPDH) was used as an internal control. Data are represented as the mean ± SD (*n* = 4). *p < 0.05 vs PBS.

### Effects of RNPs on Cell Cycle and Apoptosis

The mechanisms underlying the suppression of cell proliferation by RNPs were clarified using cell cycle and apoptosis assays. Phosphorylation of Akt, which contributes to epidermal hyperplasia and chronic inflammation promoted by M5 stimulation in HaCaT cells, was suppressed by the RNPs (Figure 5a,b). The cell cycle of M5-stimulated HaCaT cells was analyzed using flow cytometry (histograms in Figure 5c and distribution quantification in Figure 5d). M5 stimulation of HaCaT cells decreased the S phase cell proportion but increased the G2/M phase cell proportion. However, the RNPs inhibited this change in the cell cycle and maintained a cell cycle similar to that in the NT group. Furthermore, Western blotting showed that the protein levels of cyclin B1 and D1, which regulate the cell cycle in the S and G2/M phases, respectively, were decreased by the RNPs (Figure 5e,f). Figure 5g,h shows the percentage of apoptotic HaCaT cells treated with M5 and RNPs, as determined using flow cytometry and annexin V/propidium iodide staining. Actinomycin D was used as the apoptosis-inducing compound. The percentage of apoptotic M5-stimulated HaCaT cells was similar to that in the NT group, and did not change after RNP treatment. RNPs increased the proportion of G1 phase cells and decreased the proportion of S phase cells in unstimulated normal HaCaT cells (Figure S5a) but did not induce apoptosis (Figure S5b) or affect cyclin B1 and D1 protein levels (Figure S5c).

**Figure 5:**
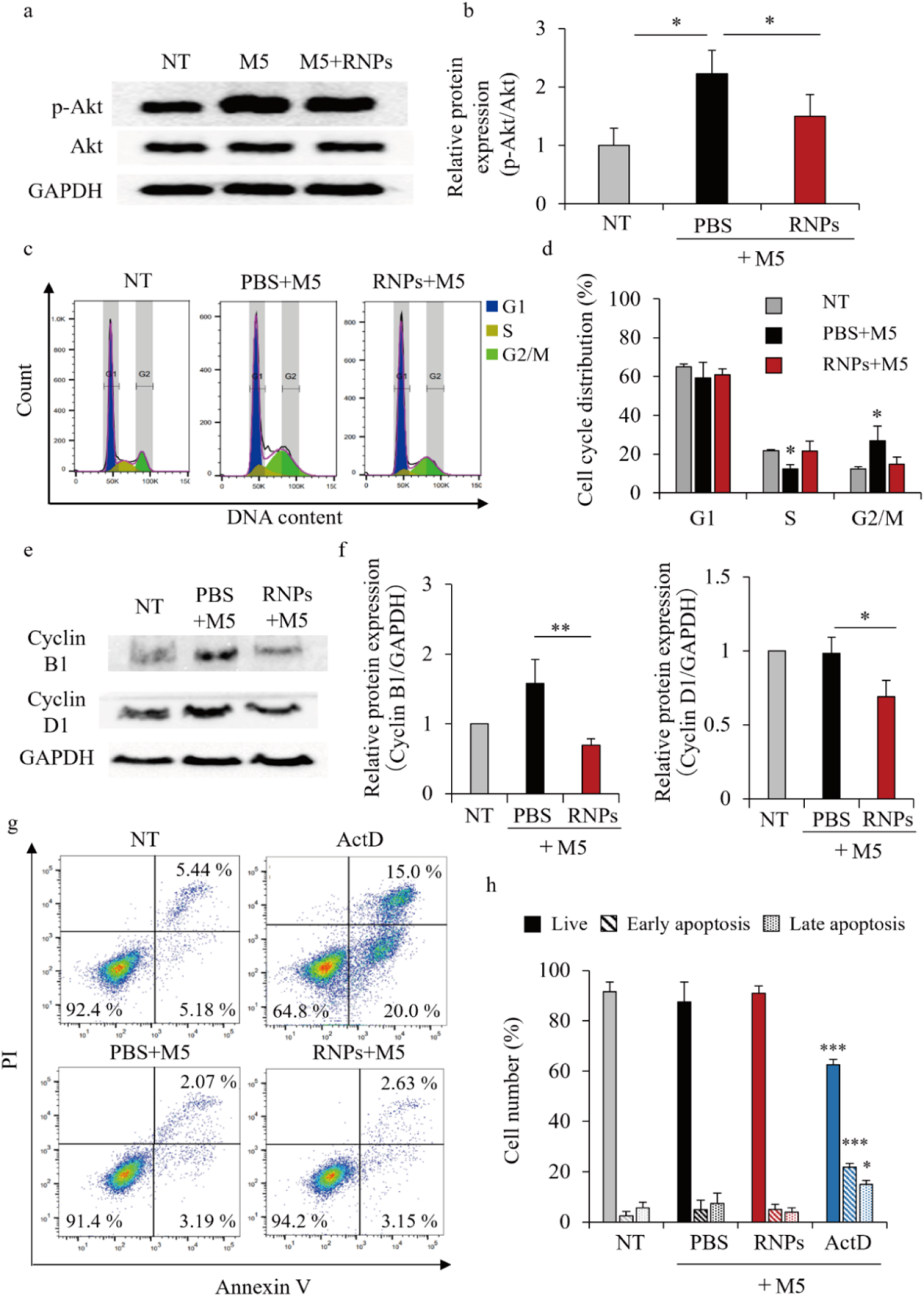
Effects of RNPs on cell cycle and apoptosis. M5-stimulated HaCaT cells were treated with 50 μg/mL of RNPs for 48 h. (a) Western blotting analysis of p-Akt and Akt expression levels. (b) Relative p-Akt and Akt expression levels normalized to GAPDH levels were analyzed using the ImageJ software. Data are represented as the mean ± SD (*n* = 3). *p < 0.05. (c) Cell cycle distribution of HaCaT (NT), M5-stimulated HaCaT (M5), and RNP-treated M5 (M5 + RNPs) cells. The cells were stained with the Cell Cycle Assay Solution Blue 48 h after treatment and analyzed using flow cytometry. (d) Percentages of cells in the G1, S, and G2/M phases determined via the FlowJo software. Data are represented as the mean ± SD (*n* = 3). *p < 0.05 vs NT. (e) Western blotting analysis of cyclin B1 and D1 expression levels. (f) Relative cyclin B1 and D1 expression levels normalized to GAPDH levels were analyzed using the ImageJ software. Data are represented as the mean ± SD (*n* = 3). *p < 0.05 and **p < 0.01. (g) Percentages of apoptotic cells in the NT, PBS + M5, RNPs + M5, and actinomycin D (ActD) groups. The cells were stained using the apoptosis detection kit and analyzed using flow cytometry. (h) Percentages of live, early apoptotic, and late apoptotic cells determined using the FlowJo software. Data are represented as the mean ± SD (*n* = 3). *p < 0.05 and ***p < 0.001.

### Effects of RNPs on Macrophages

Macrophages are important immune cell types that play a pivotal role in regulating inflammation. Therefore, the effects of RNPs on mouse macrophage-like RAW264.7 cells were evaluated. Figure 6a shows the growth of RNP-treated (0.1–100 μg/mL) RAW264.7, as determined via the cell counting kit-8 assay. The percentage of live cells did not change significantly at the tested RNP concentrations, indicating that RNPs had a minimal effect on the growth of RAW264.7 cells. Figure 6b,c shows the cellular uptake and intracellular colocalization of DiO-labeled RNPs in RAW264.7 cells after 24 h treatment, as evaluated using flow cytometry and CLSM. RNPs increased the fluorescence intensity in RAW264.7 cells in a concentration-dependent manner (Figure 6b). CLSM images revealed extensive colocalization of green RNP signals with red signals of endo/lysosomal markers in the cells (Figure 6c). Figure 6d,e shows the antioxidant activity of RNPs in inflamed RAW264.7 cells after treatment with lipopolysaccharide (LPS) for 24 h. Increased fluorescence signals of the DCFA-DA probe were observed in the LPS-stimulated RAW264.7 cells, indicating that inflammation induced ROS formation in RAW264.7 cells. However, RNPs suppressed ROS production in the LPS-stimulated RAW264.7 cells (Figure 6d). Quantification of ROS fluorescence using flow cytometry indicated that ROS levels were decreased by the RNPs in a concentration-dependent manner (Figure 6e).

**Figure 6:**
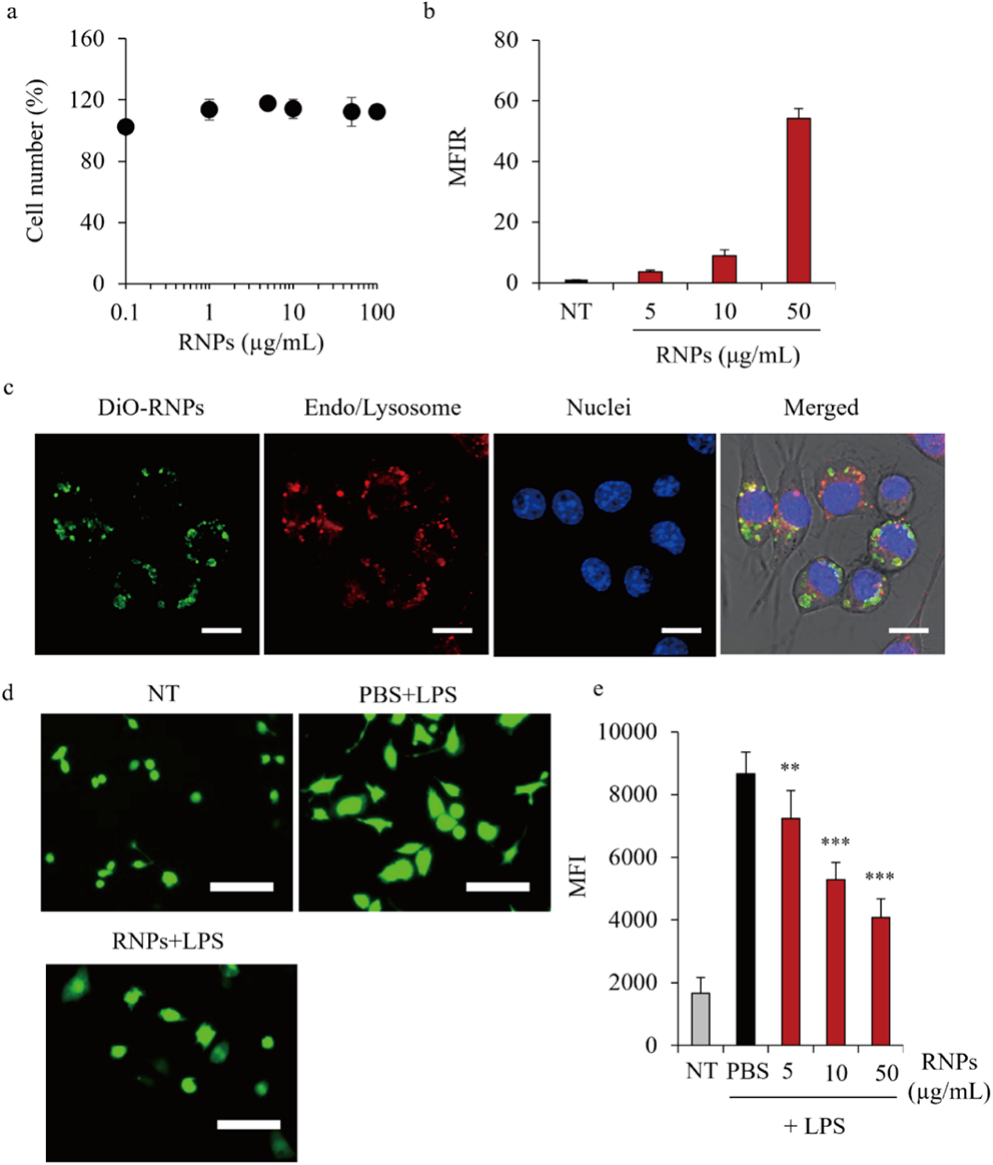
Effects of RNPs on RAW267.4 cells. (a) Number of RAW264.7 cells incubated with 0.1–100 μg/mL of RNPs at 37 °C for 6 h. Cell number was measured using the CCK-8 assay. Data are represented as the mean ± SD (*n* = 3). (b) Cellular uptake of RNPs by RAW264.7 cells incubated with 5, 10, and 50 μg/mL of DiO-RNPs at 37 °C for 6 h. Fluorescence intensity of cells was determined via flow cytometry, and cellular uptake was expressed as MFIR. Data are represented as the mean ± SD (*n* = 3). (c) Intracellular trafficking of DiO-RNPs in RAW264.7 cells. After 6 h incubation, nuclei and endosomes/lysosomes were stained with Hoechst 33342 (blue) and LysoTracker Red (red), respectively. Scale bars, 10 μm. ROS-scavenging activity of RNPs in lipopolysaccharide (LPS)-stimulated RAW264.7 cells. RAW264.7 cells stimulated with 250 ng/mL LPS were treated with RNPs for 24 h. Intracellular ROS levels were determined using the DCFH-DA dye via fluorescence microscopy (d, Scale bars, 100 μm) and flow cytometry (e). Data are represented as the mean ± SD (*n* = 3), **p < 0.01 and ***p < 0.001 vs PBS + LPS.

### Treatment of IMQ-Induced Psoriasis-Like Inflammation via Intradermal Injection of RNPs

The therapeutic efficacy of the RNPs was evaluated in a 5% IMQ-induced psoriasis-like mouse model. IMQ, a Toll-like receptor 7/8 ligand, induces dermatitis that closely resembles human psoriasis, including erythema, scaling, epidermal hyperplasia, and inflammatory cell infiltration in mice.^30,33^ RNPs were delivered to the skin via general topical application and intradermal administration of hMNs. The hMNs used in this study, MicronJet, comprise three pyramid-shaped silicon microneedles, each 600 μm in length. These microneedles have sharp tips designed to effectively penetrate the stratum corneum and epidermis, enabling controlled intradermal delivery.^34^ Figure 7a shows the distribution of RNPs in the IMQ-induced psoriatic skin after topical application or intradermal injection of hMNs. After topical application, RNPs remained localized in the stratum corneum. In contrast, hMN injection enabled efficient delivery into the upper dermis at a depth of approximately 200 μm at the puncture site (white arrow in Figure 7a). Figure 7b shows images of skin treated with RNPs for 7 days. Epidermal hyperplasia and cell infiltration induced by IMQ were significantly ameliorated by the intradermal administration of RNPs; however, topical application of RNPs had almost no effect. Epidermal thickness in normal mouse skin was approximately 10 μm, but it increased approximately 5-fold after IMQ application; however, this effect was significantly suppressed by the intradermal administration of RNPs (Figure 7c). Representative images captured on day 7 revealed that only the ID injection RNP group experienced alleviation of erythema, scaling, and thickening (Figure S6a). No significant changes in body weight were observed in any of the groups during the treatment period (Figure S6b). Notably, intravenous and subcutaneous administration were ineffective (Figure S7a,b). Therefore, the skin was thoroughly evaluated in greater detail. The mRNA levels of *KRT6* (Figure 7d) and Ki-67 (Figure 7e), nuclear proteins widely used as markers of cellular proliferation, were significantly decreased by intradermal administration of RNPs to the skin at the lesion site. The mRNA levels of *TNF-α* and *IL-1β*, two prototypical pro-inflammatory cytokines, were also significantly decreased by the intradermal administration of RNPs (Figure 7f). In addition, immunofluorescence staining revealed increased HO-1 expression in the epidermis of IMQ-treated skin, which was suppressed by intradermal RNP administration (Figure 7g). Figure 7h shows the effects of RNPs on immune cell infiltration via immunostaining. The expression levels of F4/80, a macrophage marker, were reduced by the RNPs, whereas those of Ly6*G*/6C, a neutrophil and monocyte marker, remained unchanged.

**Figure 7:**
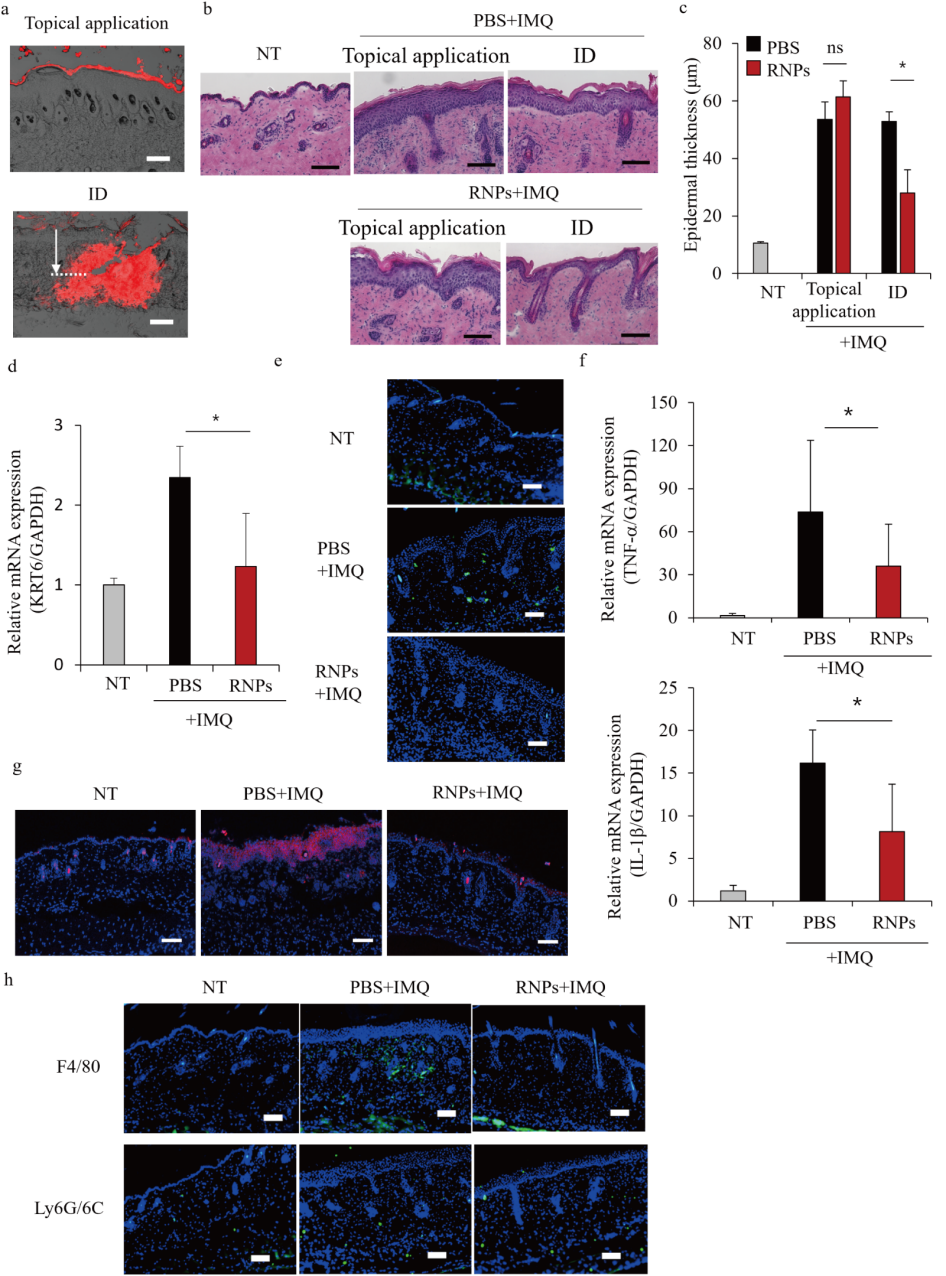
Treatment of imiquimod (IMQ)-induced psoriasis-like inflammation model mice via intradermal injection of RNPs. (a) Distribution of DiI-labeled RNPs immediately after topical application (1 h postinjection) and intradermal (ID) injection using hollow microneedles (hMNs) (immediately after application). The white arrow indicates the depth of needle injection. Scale bars, 100 μm. (b) Histological analysis of RNP-treated psoriatic skin in IMQ-induced psoriasis model mice. RNPs were administered via intradermal injection (ID) and topical application (100 μg/mL) for seven consecutive days. Skin samples were stained with hematoxylin and eosin (H&E). Scale bars, 100 μm. (c) Quantification of epidermal thickness in RNP-treated psoriatic skin using the BZ-X Analyzer imaging software. Data are represented as the mean ± SD (*n* = 4). *p < 0.05; ns, not significant. (d) *KRT6* mRNA levels in the skin quantified using RT-qPCR. Data are represented as the mean ± SD (*n* = 4). *p < 0.05. (e) Representative images of the immunofluorescence staining for Ki-67 (green). Cell nuclei were stained with 4’,6-diamidino-2-phenylindole (DAPI; blue). Scale bars, 100 μm. (f) Tumor necrosis factor (*TNF*)-α and *IL-1β* mRNA levels in the skin quantified using RT-qPCR. Data are represented as the mean ± SD (*n* = 4). *p < 0.05. (g) Skin sections were collected from healthy skin (no treatment; NT), IMQ + PBS-treated, and IMQ + RNP-treated mice on day 7 and subjected to HO-1 staining (red). Nuclei were counterstained with DAPI (blue). Scale bars, 100 μm. (h) Representative images of the immunofluorescence staining for macrophage marker F4/80 and neutrophil marker Ly6*G*/6C (green) observed via CLSM. Cell nuclei were stained with DAPI (blue). Scale bars, 100 μm.

## Discussion

Recent research on animal-derived exosomes has highlighted their potential use as therapeutic agents and biomarkers.^35^ However, their clinical application remains limited due to challenges such as high cost, low yield, and immunogenicity. Bacterial extracellular vesicles are being actively investigated for use in immunotherapy and vaccine development.^36^ However, their high immunogenicity may restrict their applicability in contexts that require immune tolerance and biocompatibility. In contrast, pdNPs are safer and more scalable alternatives that offer unique advantages, particularly for the treatment of inflammatory disorders. They can be obtained from edible medicinal plants, are generally recognized as safe, and exhibit low immunogenicity.^37^ Additionally, they are naturally enriched in anti-inflammatory and antioxidant phytochemicals, which may help suppress inflammation, oxidative stress, and barrier dysfunction associated with skin pathology. Given these advantages, the interest in pdNPs has increased significantly in recent years. Improving the purity of plant-derived EVs is important for their future applications. Mechanical disruption, which is commonly used for isolation, may cause contamination with other membrane components. Although affinity purification using EV markers, such as TET8, has been reported in *Arabidopsis*,^38^ limited information in other species hinders its broad applicability. The development of broadly applicable purification methods for a diverse range of plants remains a key challenge.

To the best of our knowledge, this is the first study to identify nanoparticles from rose hips, a medicinal plant with multiple benefits. The physicochemical properties of the RNPs, including their 100–200 nm particle size distribution and lipid bilayer structure (Figure 1a–c and Table 1), suggest exosome-like properties. Notably, the lipid composition of the RNPs differed from that of the rose hip lipid extract, with PE enrichment (Figure S1) and increased saturated fatty acid levels (Figure 1e). These findings suggest that the lipid constituents of the RNPs may contribute to their biological activity. Although proteomic analyses can provide further insights, the lack of well-annotated protein databases for nonmodel plant species limits the reliability of such analyses.^39^ Further untargeted lipidomics and improved proteomics may help to clarify the bioactive components of pdNPs. Our results indicate that the RNP composition differed from that of rose hip fruit. Lipidomic analysis revealed distinct phospholipid classes in PC-3 cells and exosomes.^40^ Exosomes exhibit higher saturated fatty acid levels than cells.^41^ This may be related to exosomal membrane stability because lipid unsaturation contributes to membrane fluidity.^42,43^ These reports suggest that selective lipid incorporation and remodeling during nanoparticle formation support the structural stability and unique biological functions of RNPs.

Plants have evolved antioxidant defense systems to protect themselves from excessive oxidative stress. An important feature of pdNPs is the abundance of antioxidant components.^44^ In this study, RNPs exhibited antioxidant activity by scavenging ROS (Figure 3a–c). Ascorbic acid, β-carotene, and gallic acid, which are abundant in rose hips,^45,46^ were quantitatively analyzed. Ascorbic acid, the most abundant component in rose hips, was barely incorporated into RNPs, whereas β-carotene and gallic acid were detected in sufficient amounts (Figure 1d). This suggests that hydrophobic compounds are more easily incorporated into particles with lipid bilayer structures than are hydrophilic compounds. Because ginger-derived nanoparticles contain high concentrations of 6-gingerol and 6-shogaol,^16^ pdNPs may also contain hydrophobic compounds. Both β-carotene and gallic acid, which were detected in RNPs, exhibit strong antioxidant activity. These antioxidants may contribute to the therapeutic effects, as gallic acid modulates inflammation via the Nrf2 pathway,^47^ and β-carotene, a vitamin A precursor, supports skin homeostasis and immune regulation. In particular, the clinical trial using 9-cis β-carotene-rich powder demonstrated improvement in psoriasis conditions, supporting the relevance of β-carotene-related compounds in psoriasis treatment.^48^ These findings suggest that selective encapsulation of hydrophobic bioactive components may contribute to the antipsoriatic effects of RNPs. In addition, RNP treatment suppressed H_2_O_2_-induced Nrf2 phosphorylation and HO-1 expression in HaCaT cells (Figure 3h,i), suggesting that RNPs reduce oxidative stress through direct ROS scavenging rather than by activating the Nrf2 pathway. Similarly, in vivo staining revealed reduced HO-1 expression in IMQ-induced psoriatic skin after RNP treatment (Figure 7g), suggesting that RNPs exert antioxidant effects without activating the Nrf2–HO-1 pathway.

Among the tested skin-related cells, the RNPs were efficiently taken up by HaCaT cells (Figure 2a,b), and the same trend was observed for cell proliferation inhibition (Figure S2). Additionally, pretreatment with RNPs followed by medium washout suppressed both intracellular ROS production and M5-induced hyperproliferation (Figures 3d–g and S4c), suggesting that internalized RNPs mediate these effects. These findings suggest that the cellular uptake of RNPs is important for their function in keratinocytes. Their high selectivity for keratinocytes, which are targets of psoriasis pathogenesis, highlights their efficacy and safety. The addition of RNPs to M5-stimulated HaCaT cells significantly suppressed hyperproliferation (Figure 4a–c) caused by Akt signaling-mediated phosphorylation and cell cycle arrest (Figure 5). The Akt signaling pathway is a potential therapeutic target for psoriasis.^49^ Abnormal lipid metabolism is also important in the pathogenesis of psoriasis, and changes in epidermal lipid levels reported in affected patients.^50^ miRNAs are also involved in the inflammation and proliferation of epidermal cells.^51,52^ MiRNAs and lipids in RNPs may ameliorate the abnormal state of epidermal cells, warranting further investigation.

RNPs were efficiently taken up by RAW264.7, and they suppressed intracellular ROS production (Figure 6). PS is readily recognized by macrophages as a phagocytic signal^53,54^ and PS liposomes are used as drug delivery systems to target macrophages.^55^ The effects of PS-containing RNPs on RAW264.7 cells were not unexpected. Although RNP uptake increased dose-dependently in RAW264.7 cells (Figure 6b), ROS suppression plateaued (Figure 6e). This discrepancy may reflect the saturation of antioxidant activity (Figure 3a,b) and limited access of RNPs to mitochondria-derived ROS due to endosomal confinement (Figure 6c). The abnormal state of keratinocytes in psoriasis is closely associated with immune cells. The effects of RNPs on keratinocytes and macrophages render them excellent pdNPs with multiple therapeutic mechanisms. DiI-labeled RNPs showed widespread distribution after intradermal injection (Figure 7a), making it difficult to identify specific target cells. These cells are likely involved; however, further confirmation is required. Future analyses using enzymatically isolated skin cells and flow cytometry may clarify cellular uptake.

Because of their liposome-like structures, pdNPs are promising transdermal delivery carriers for skin diseases. Previous studies have shown the skin penetration of hydrophobic and hydrophilic fluorescent dyes using cucumber^56^- and broccoli^57^-derived nanoparticles, highlighting pdNPs as effective skin nanocarriers. However, the topical application of RNPs did not show efficacy in this study (Figure 7a,b). Various lipid-based nanocarriers have been developed to improve skin penetration, and membrane fluidity plays an important role in determining the extent of the nanocarriers.^7,58^ RNPs are rich in saturated fatty acids that confer stable membrane properties and limit skin permeability. Their poor permeability is also evidenced by their accumulation in the stratum corneum after topical application, as observed in psoriatic skin (Figure 7a), and may contribute to reduced therapeutic efficacy. In addition, the limited effects observed with the IV and SC routes may be attributed to systemic dilution, rapid clearance, or insufficient delivery to the affected skin. Therefore, RNPs should be combined with suitable intradermal delivery agents, such as the hMNs used in this study.

## Conclusion

In this study, negatively charged RNPs with lipid bilayer structures were isolated from rose hips. The RNPs exhibited antioxidant activity, suppressed excessive keratinocyte proliferation, and downregulated inflammatory cytokine levels, indicating their therapeutic potential for psoriasis by modulating oxidative stress and epidermal proliferation pathways. Furthermore, the RNPs were taken up by macrophages and exhibited intracellular ROS-scavenging activity. Intradermal injection of RNPs improved psoriasis-like skin lesions in model mice in vivo, highlighting the importance of targeted drug delivery for the treatment of psoriasis. Overall, this study outlines a promising strategy combining RNPs, which exert potent effects on epidermal cells, with hMNs, which facilitate precise epidermal drug delivery for psoriasis treatment.

## Supplementary Material



## Notes

Lipid composition of RNPs (Figure S1); effects of RNPs on A375, HDFa, and HaCaT cells (Figure S2); uptake pathway of DiO-labeled RNPs in HaCaT cells (Figure S3); inhibitory effects of RNPs, PS-liposomes, and calcitriol on M5 cytokine-induced keratinocyte proliferation (Figure S4); cell cycle, apoptosis, and cyclin expression in RNP-treated HaCaT cells (Figure S5); therapeutic effects of RNPs in IMQ-induced psoriatic mice by topical or intradermal administration (Figure S6); histological analysis and epidermal thickness quantification in RNP-treated psoriatic mice (Figure S7); siRNA sequences (Table S1); primer sequences for RT-qPCR (Table S2) (PDF)

## References

[1] Zhou X., Chen Y., Cui L., Shi Y., Guo C. (2022). Advances in
the pathogenesis of psoriasis: from keratinocyte perspective. Cell Death Dis..

[2] Liu S., He M., Jiang J., Duan X., Chai B., Zhang J., Tao Q., Chen H. (2024). Triggers for the onset and recurrence of psoriasis:
a review and update. Cell Commun. Signaling.

[3] Guo J., Zhang H., Lin W., Lu L., Su J., Chen X. (2023). Signaling pathways and targeted therapies
for psoriasis. Signal Transduction Targeted
Ther..

[4] Cannavò S. P., Riso G., Casciaro M., Di Salvo E., Gangemi S. (2019). Oxidative
stress involvement in psoriasis: a systematic review. Free Radical Res..

[5] Pleńkowska J., Gabig-Cimińska M., Mozolewski P. (2020). Oxidative
stress as an important contributor to the pathogenesis of psoriasis. Int. J. Mol. Sci..

[6] Hu J., Bian Q., Ma X., Xu Y., Gao J. (2022). A double-edged
sword: ROS related therapies in the treatment of psoriasis. Asian J. Pharm. Sci..

[7] Xu J., Chen H., Qian H., Wang F., Xu Y. (2022). Advances in
the modulation of ROS and transdermal administration for anti-psoriatic
nanotherapies. J. Nanobiotechnol..

[8] Ahmed S. S., Manchanda Y., De A., Das S., Kumar R. (2023). Topical therapy
in psoriasis. Indian J. Dermatol..

[9] Kumar M. A., Baba S. K., Sadida H. Q., Marzooqi S. A., Jerobin J., Altemani F. H., Algehainy N., Alanazi M. A., Abou-Samra A.-B., Kumar R. (2024). Extracellular
vesicles as tools and targets in therapy
for diseases. Signal Transduction Targeted Ther..

[10] Langellotto M. D., Rassu G., Serri C., Demartis S., Giunchedi P., Gavini E. (2025). Plant-derived extracellular
vesicles: a synergetic
combination of a drug delivery system and a source of natural bioactive
compounds. Drug Delivery Transl. Res..

[11] Feng J., Xiu Q., Huang Y., Troyer Z., Li B., Zheng L. (2023). Plant-derived
vesicle-like nanoparticles as promising biotherapeutic tools: present
and future. Adv. Mater..

[12] Welsh J. A., Goberdhan D. C. I., O’Driscoll L., Buzas E. I., Blenkiron C., Bussolati B., Cai H., Di Vizio D., Driedonks T. A. P., Erdbrügger U. (2024). Minimal information
for studies of extracellular vesicles (MISEV2023): from basic to advanced
approaches. J. Extracell. Vesicles.

[13] Azizi F., Kazemipour-Khabbazi S., Raimondo S., Dalirfardouei R. (2024). Molecular
mechanisms and therapeutic application of extracellular vesicles from
plants. Mol. Biol. Rep..

[14] Pinedo M., de la Canal L., de Marcos Lousa C. (2021). A call for
Rigor and standardization
in plant extracellular vesicle research. J.
Extracell. Vesicles.

[15] Chen X., Xing X., Lin S., Huang L., He L., Zou Y., Zhang X., Su B., Lu Y., Zheng D. (2023). Plant-derived
nanovesicles: harnessing nature’s power for tissue protection
and repair. J. Nanobiotechnol..

[16] Zhang M., Viennois E., Prasad M., Zhang Y., Wang L., Zhang Z., Han M. K., Xiao B., Xu C., Srinivasan S., Merlin D. (2016). Edible ginger-derived nanoparticles:
A novel therapeutic approach for the prevention and treatment of inflammatory
bowel disease and colitis-associated cancer. Biomaterials.

[17] Sasaki D., Kusamori K., Takayama Y., Itakura S., Todo H., Nishikawa M. (2021). Development
of nanoparticles derived from corn as mass
producible bionanoparticles with anti-cancer activity. Sci. Rep..

[18] Sasaki D., Suzuki H., Kusamori K., Itakura S., Todo H., Nishikawa M. (2024). Development
of rice bran-derived nanoparticles with
excellent anti-cancer activity and their application for peritoneal
dissemination. J. Nanobiotechnol..

[19] El
Maghraby G. M., Barry B. W., Williams A. C. (2008). Liposomes and skin:
from drug delivery to model membranes. Eur.
J. Pharm. Sci..

[20] Jafari A., Daneshamouz S., Ghasemiyeh P., Mohammadi-Samani S. (2023). Ethosomes
as dermal/transdermal drug delivery systems: applications, preparation
and characterization. J. Liposome Res..

[21] Jain S., Jain P., Umamaheshwari R. B., Jain N. K. (2003). Transfersomes—a
novel vesicular carrier for enhanced transdermal delivery: development,
characterization, and performance evaluation. Drug Dev. Ind. Pharm..

[22] Guillot A. J., Martínez-Navarrete M., Garrigues T. M., Melero A. (2023). Skin drug delivery using lipid vesicles:
A starting
guideline for their development. J. Controlled
Release.

[23] Nowak-Perlak M., Szpadel K., Jabłońska I., Pizon M., Woźniak M. (2022). Promising strategies in plant-derived treatments of
psoriasis-update of in vitro, in vivo, and clinical trials studies. Molecules.

[24] Kaur A., Kumar S. (2012). Plants and plant products with potential
anti-psoriatic activity—a
review. Pharm. Biol..

[25] Gavra D. I., Endres L., Pető Á., Józsa L., Fehér P., Ujhelyi Z., Pallag A., Marian E., Vicas L. G., Ghitea T. C. (2022). In
vitro and human pilot
studies of different topical formulations containing Rosa species
for the treatment of psoriasis. Molecules.

[26] Gupta S., Rawat S., Arora V., Kottarath S. K., Dinda A. K., Vaishnav P. K., Nayak B., Mohanty S. (2018). An improvised
one-step sucrose cushion ultracentrifugation method for exosome isolation
from culture supernatants of mesenchymal stem cells. Stem Cell Res. Ther..

[27] Yajima K., Chiba S., Park I., Ogata H., Kayaba M., Ishihara A., Tanaka Y., Simeng Z., Jaehoon S., Katakura M., Tokuyama K. (2024). Dietary palmitic
acid to oleic acid
ratio modulates energy metabolism and biological rhythms in young
healthy Japanese males. Br. J. Nutr..

[28] Itakura S., Shohji A., Amagai S., Kitamura M., Takayama K., Sugibayashi K., Todo H. (2023). Gene knockdown in HaCaT cells by
small interfering RNAs entrapped in grapefruit-derived extracellular
vesicles using a microfluidic device. Sci. Rep..

[29] Guilloteau K., Paris I., Pedretti N., Boniface K., Juchaux F., Huguier V., Guillet G., Bernard F. X., Lecron J. C., Morel F. (2010). Skin inflammation induced
by the synergistic action of IL-17A, IL-22,
oncostatin M, IL-1alpha, and TNF-alpha recapitulates some features
of psoriasis. J. Immunol..

[30] van
der Fits L., Mourits S., Voerman J. S. A., Kant M., Boon L., Laman J. D., Cornelissen F., Mus A. M., Florencia E., Prens E. P., Lubberts E. (2009). Imiquimod-induced
psoriasis-like skin inflammation in mice is mediated via the IL-23/IL-17
axis. J. Immunol..

[31] Gao J., Chen F., Fang H., Mi J., Qi Q., Yang M. (2020). Daphnetin inhibits proliferation
and inflammatory response in human
HaCaT keratinocytes and ameliorates imiquimod-induced psoriasis-like
skin lesion in mice. Biol. Res..

[32] Yin X., Yang Z., Zhu M., Chen C., Sun Q. (2022). Role of the
long non-coding RNA, SPRR2C, based on an in vitro psoriatic keratinocyte
cell model. Eur. J. Dermatol..

[33] Xiong Y., Wang J., Wang S., Li H., Zhou X. (2023). Tryptanthrin
ameliorates imiquimod-induced psoriasis in mice by suppressing inflammation
and oxidative stress via NF-kappaB/MAPK/Nrf2 pathways. J. Nat. Med..

[34] Levin Y., Kochba E., Hung I., Kenney R. (2015). Intradermal
vaccination
using the novel microneedle device MicronJet600: past, present, and
future. Hum. Vaccines Immunother..

[35] Fusco C., De Rosa G., Spatocco I., Vitiello E., Procaccini C., Frigè C., Pellegrini V., La Grotta R., Furlan R., Matarese G. (2024). Extracellular vesicles
as human therapeutics: A scoping review of the literature. J. Extracell. Vesicles.

[36] Muñoz-Echeverri L. M., Benavides-López S., Geiger O., Trujillo-Roldán M. A., Valdez-Cruz N. A. (2024). Bacterial extracellular vesicles: biotechnological
perspective for enhanced productivity. World
J. Microbiol. Biotechnol..

[37] Zhao Y., Tan H., Zhang J., Pan B., Wang N., Chen T., Shi Y., Wang Z. (2023). Plant-derived
vesicles: A New Era for anti-cancer drug
delivery and cancer treatment. Int. J. Nanomed..

[38] Huang Y., Wang S., Cai Q., Jin H. (2021). Effective methods for
isolation and purification of extracellular vesicles from plants. J. Integr. Plant Biol..

[39] Carpentier S. C., Panis B., Vertommen A., Swennen R., Sergeant K., Renaut J., Laukens K., Witters E., Samyn B., Devreese B. (2008). Proteome analysis of
non-model plants: a challenging
but powerful approach. Mass Spectrom. Rev..

[40] Llorente A., Skotland T., Sylvänne T., Kauhanen D., Róg T., Orłowski A., Vattulainen I., Ekroos K., Sandvig K. (2013). Molecular
lipidomics of exosomes released by PC-3 prostate cancer cells. Biochim. Biophys. Acta.

[41] Trajkovic K., Hsu C., Chiantia S., Rajendran L., Wenzel D., Wieland F., Schwille P., Brügger B., Simons M. (2008). Ceramide triggers budding
of exosome vesicles into multivesicular endosomes. Science.

[42] Maulucci G., Cohen O., Daniel B., Sansone A., Petropoulou P. I., Filou S., Spyridonidis A., Pani G., De Spirito M., Chatgilialoglu C. (2016). Fatty acid-related modulations of membrane
fluidity in cells: detection and implications. Free Radical Res..

[43] Leekumjorn S., Cho H. J., Wu Y., Wright N. T., Sum A. K., Chan C. (2009). The role of fatty acid
unsaturation in minimizing biophysical changes
on the structure and local effects of bilayer membranes. Biochim. Biophys. Acta.

[44] Kim M., Jang H., Kim W., Kim D., Park J. H. (2023). Therapeutic
applications of plant-derived extracellular vesicles as antioxidants
for oxidative stress-related diseases. Antioxidants.

[45] Jimenez S., Gascon S., Luquin A., Laguna M., Ancin-Azpilicueta C., Rodriguez-Yoldi M. J. (2016). Rosa canina
Extracts have antiproliferative and antioxidant
effects on Caco-2 human colon cancer. PLoS One.

[46] Zhou M., Sun Y., Luo L., Pan H., Zhang Q., Yu C. (2023). Road to a
bite of rosehip: A comprehensive review of bioactive compounds, biological
activities, and industrial applications of fruits. Trends Food Sci. Technol..

[47] Zhang J., Li X., Wei J., Chen H., Lu Y., Li L., Han L., Lu C. (2018). Gallic acid inhibits the expression of keratin 16 and
keratin 17 through Nrf2 in psoriasis-like skin disease. Int. Immunopharmacol..

[48] Greenberger S., Harats D., Salameh F., Lubish T., Harari A., Trau H., Shaish A. (2012). 9-cis-rich β-carotene
powder
of the alga Dunaliella reduces the severity of chronic plaque psoriasis:
a randomized, double-blind, placebo-controlled clinical trial. J. Am. Coll. Nutr..

[49] Niu M., Li X., Li M., Chen F., Cao H., Liu Q., Liang B., Pan G., Liang C., Gao J. (2025). Curcumol attenuates
hyperproliferation and inflammatory response in a psoriatic HaCaT
keratinocyte model by inhibiting the PI3K-Akt pathway and ameliorates
skin lesions and inflammatory status in psoriasis-like mice. Inflammopharmacology.

[50] Tsambaos D., Mahrle G. (1979). The phospholipid pattern
in the involved and the uninvolved
psoriatic epidermis. Arch. Dermatol. Res..

[51] Li M., Ding Y., Tuersong T., Chen L., Zhang M.-L., Li T., Feng S.-M., Guo Q. (2024). Let-7 family regulates HaCaT cell
proliferation and apoptosis via the DeltaNp63/PI3K/AKT pathway. Open Med..

[52] Shahine Y., El-Aal S. A. A., Reda A. M., Sheta E., Atia N. M., Abdallah O. Y., Ibrahim S. S. A. (2023). Diosmin
nanocrystal gel alleviates
imiquimod-induced psoriasis in rats via modulating TLR7,8/NF-kappaB/micro
RNA-31, AKT/mTOR/P70S6K milieu, and Tregs/Th17 balance. Inflammopharmacology.

[53] Naeini M. B., Bianconi V., Pirro M., Sahebkar A. (2020). The role of phosphatidylserine
recognition receptors in multiple biological functions. Cell. Mol. Biol. Lett..

[54] Segawa K., Kurata S., Yanagihashi Y., Brummelkamp T. R., Matsuda F., Nagata S. (2014). Caspase-mediated cleavage
of phospholipid
flippase for apoptotic phosphatidylserine exposure. Science.

[55] Zhang L., Li Y., Liu X., He X., Zhang J., Zhou J., Qiao Y., Wu H., Sun F., Zhou Q. (2024). Optimal development
of apoptotic cells-mimicking liposomes targeting macrophages. J. Nanobiotechnol..

[56] Abraham A. M., Wiemann S., Ambreen G., Zhou J., Engelhardt K., Brüßler J., Bakowsky U., Li S. M., Mandic R., Pocsfalvi G., Keck C. M. (2022). Cucumber-derived exosome-like vesicles
and PlantCrystals for improved dermal drug delivery. Pharmaceutics.

[57] Yepes-Molina L., Martínez-Ballesta M. C., Carvajal M. (2020). Plant plasma
membrane
vesicles interaction with keratinocytes reveals their potential as
carriers. J. Adv. Res..

[58] Jijie R., Barras A., Boukherroub R., Szunerits S. (2017). Nanomaterials
for transdermal drug delivery: beyond the state of the art of liposomal
structures. J. Mater. Chem. B.

