# Supplementary material for: Therapeutic Potential of Rose Hip-Derived Nanoparticles for Psoriatic Skin Inflammation
Source: ACS Biomater Sci Eng. 2025 Sep 26;11(10):5938–51. doi: 10.1021/acsbiomaterials.5c00826 (PMC12522095; doi:10.1021/acsbiomaterials.5c00826)
Supplement: Supplementary file 1 [file ab5c00826_si_001.pdf]

Supporting Information

Therapeutic potential of rose hip-derived nanoparticles for psoriatic skin inflammation

Masahiro Hashimoto[a,‡], Shoko Itakura[a,‡,], Kosuke Kusamori[a], Katsuhiko Yajima[b], Shota Mitsuhashi[b], Shinichiro Hayashi[c], Hiroaki Todo[b], and Makiya Nishikawa[a]*

[a]Faculty of Pharmaceutical Sciences, Tokyo University of Science, 6-3-1 Niijuku, Katsushika, Tokyo 125-8585, Japan

[b]Faculty of Pharmacy and Pharmaceutical Sciences, Josai University, 1-1 Keyakidai, Sakado, Saitama 350-0295, Japan

[c]Green Flask Laboratory, 1-25-1 Jiyugaoka, Meguro-ku, Tokyo 152-0035, Japan

[‡]These authors equally contributed to this work.

[*]Corresponding author:

Shoko Itakura, PhD

Faculty of Pharmaceutical Sciences, Tokyo University of Science, 6-3-1 Niijuku, Katsushika, Tokyo 125-8585, Japan

E-mail: itakura@rs.tus.ac.jp

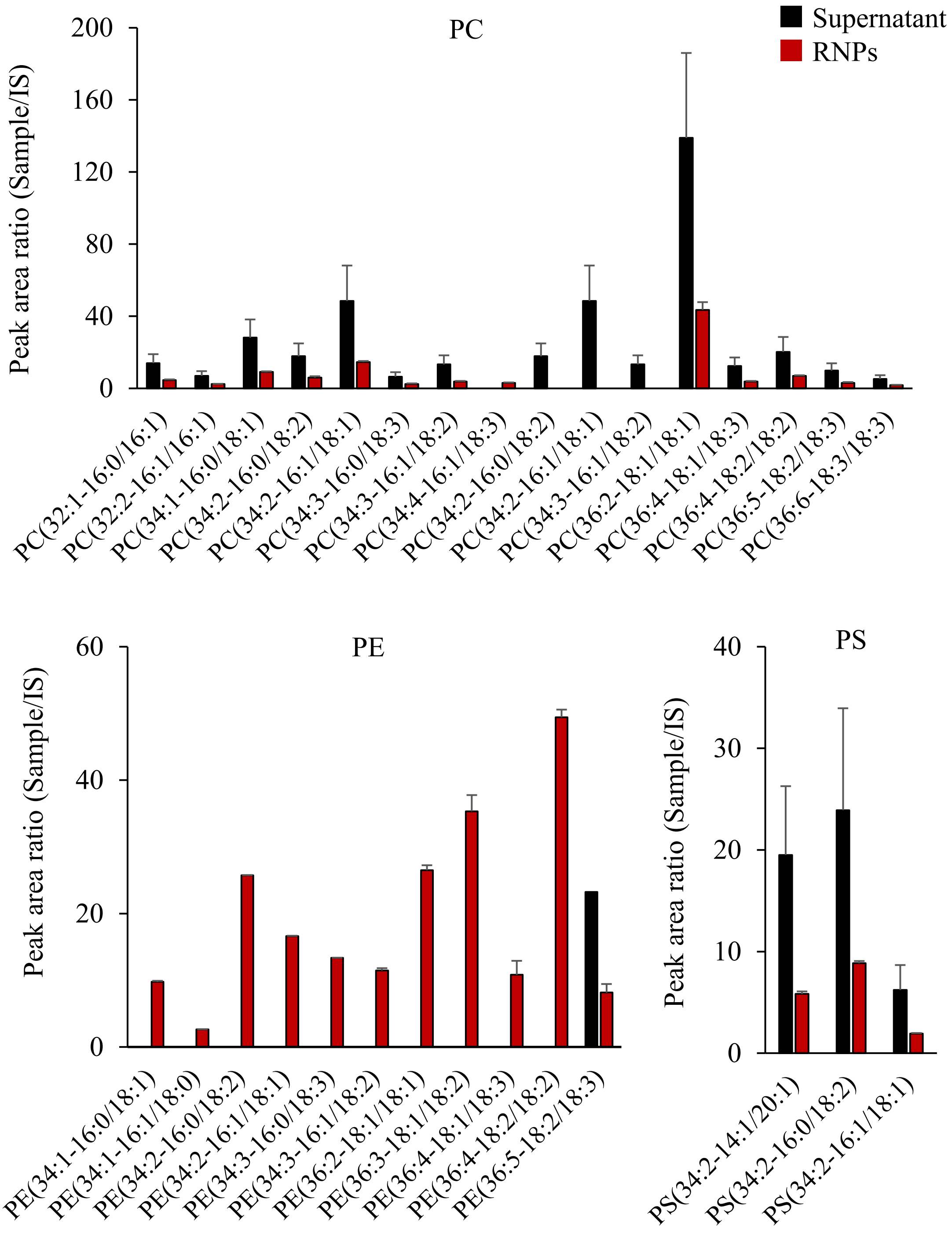

Figure S1. Lipid composition of the rose hip-derived nanoparticles (RNPs). Phosphatidylcholine (PC), phosphatidylethanolamine (PE), and phosphatidylserine (PS) levels in RNPs were determined via liquid chromatography-tandem mass spectrometry (LC-MS/MS). Data are represented as the mean ± SD (n = 3).

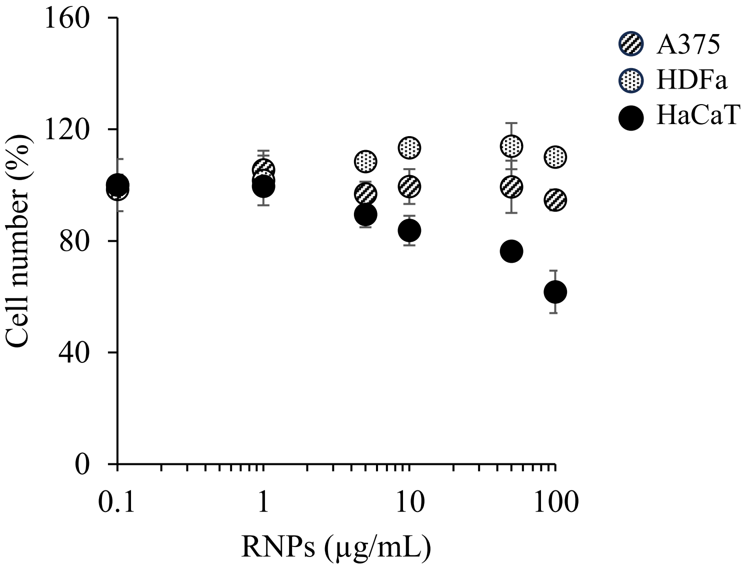

Figure S2. Effects of RNPs on A375, HDFa, and HaCaT cell numbers. The cells were incubated with 0.1–100 µg/mL RNPs at 37 °C for 48 h, and cell numbers were measured via cell counting kit (CCK)-8 assay. Data are represented as the mean ± SD ($n = 3$).

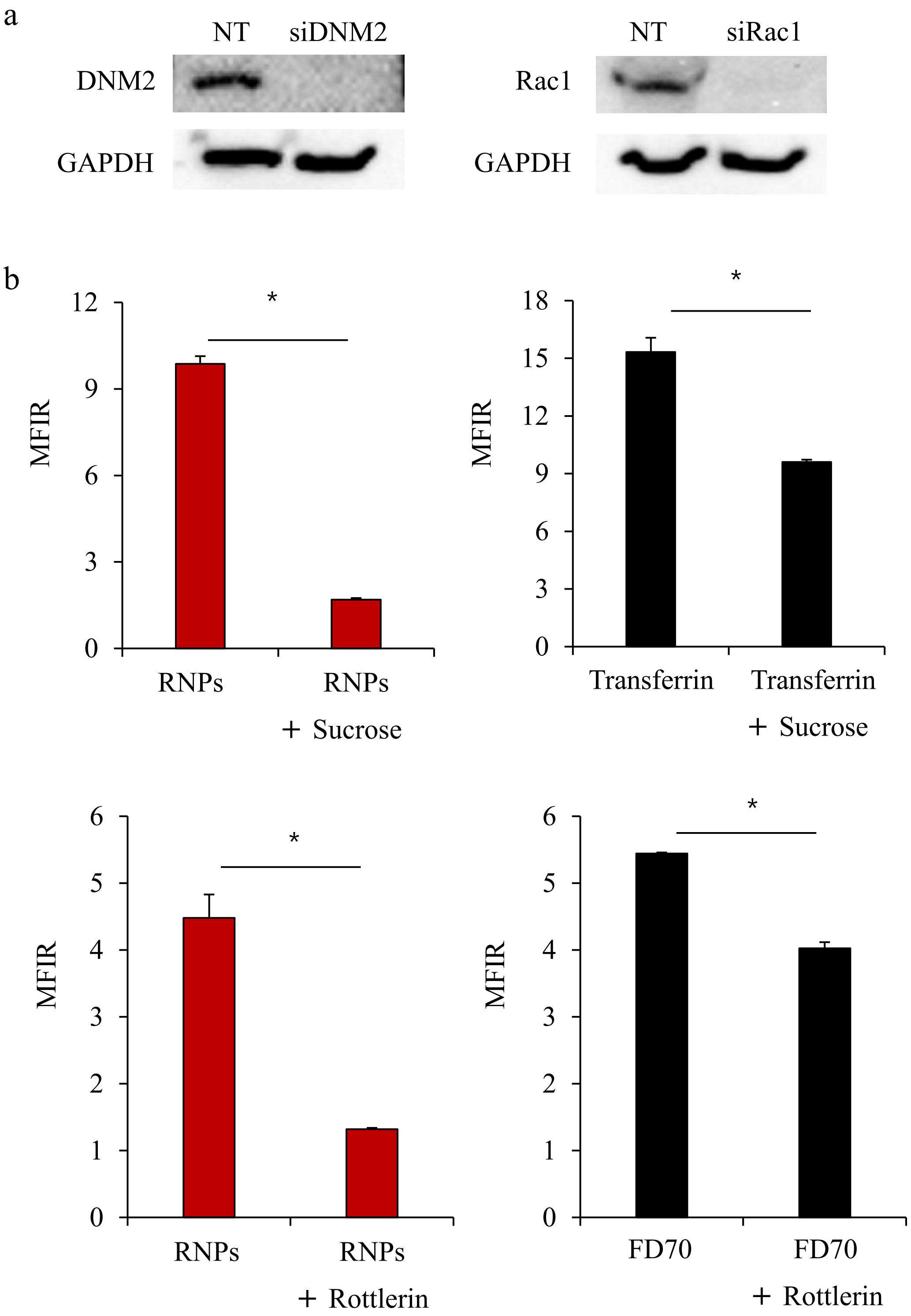

Figure S3. Quantitative flow cytometric analysis of DiO-labeled RNPs internalized by HaCaT cells. (a) Western blotting analysis of DNM2 and Rac1 expression levels following siRNA-mediated knocldown. (b) Clathrin-mediated endocytosis inhibitor (0.4 M sucrose) and micropinocytosis inhibitor (5 μM rottlerin) were added to HaCaT cells and incubated for 30 min. The cells were incubated with 3,3′-dioctadecyloxacarbocyanine (DiO)-labeled RNPs for 6 h at 37 °C. Fluorescein isothiocyanate (FITC)-labeled transferrin (20 μg/mL) and FD70 (500 μg/mL) were taken up via clathrin-mediated endocytosis and macropinocytosis, respectively. Mean fluorescence intensity ratio (MFIR) of RNPs in HaCaT cells was quantified via flow cytometry. Data are represented as the mean ± SD ($n = 3$). *$p < 0.05$.

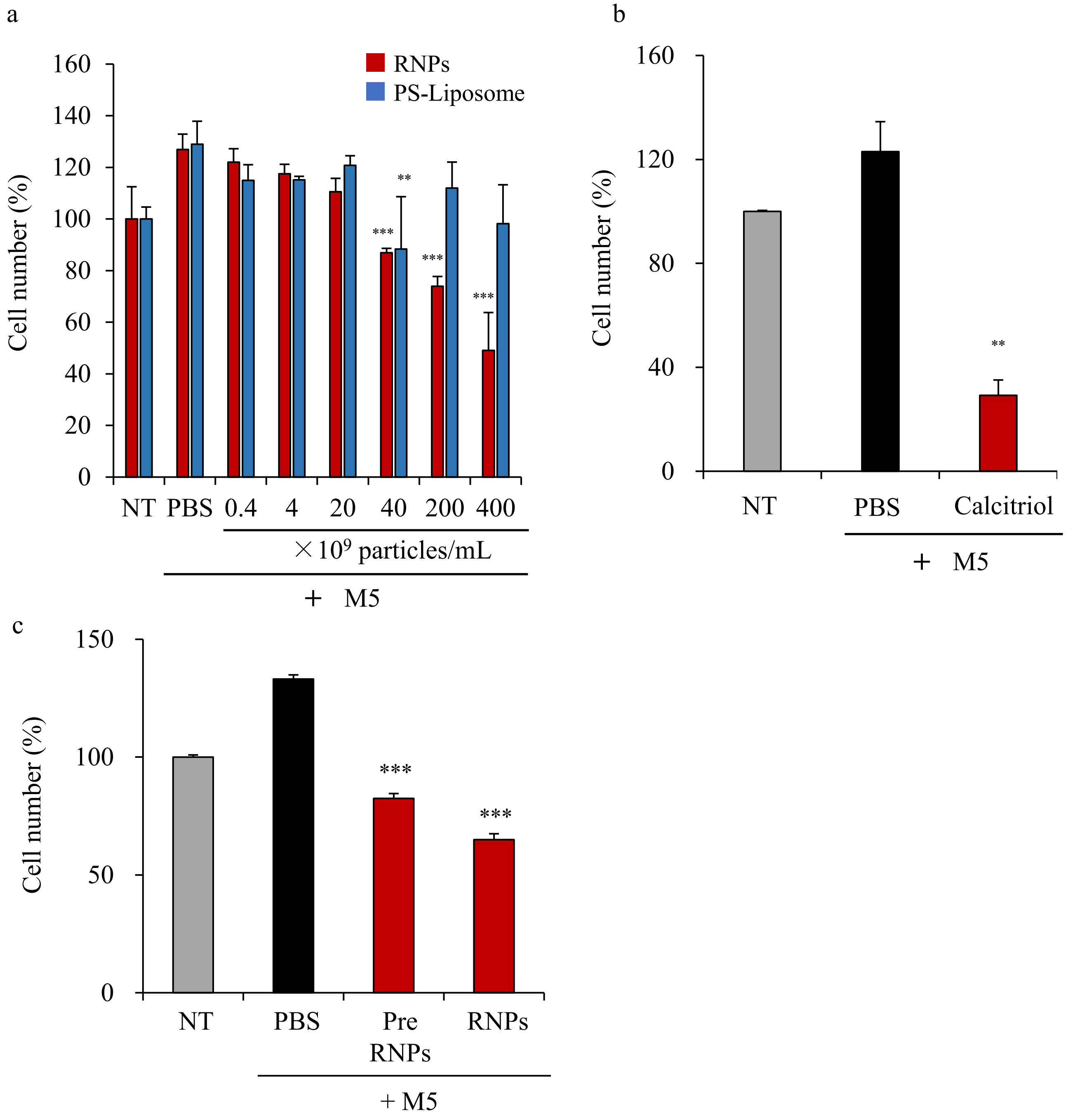

Figure S4. (a) Effects of RNPs, PS-liposome on HaCaT cell number. HaCaT cells were incubated with 0.4 - 400 $\times 10^9$ particles/mL RNPs or PS-liposome in the presence of M5 cytokines for 48 h. PS-liposomes were added at a particle number equivalent to that of RNPs. (b) HaCaT cells were treated with 10 nM calcitriol as a positive control in the presence of M5 cytokines for 48 h. (c) HaCaT cells were preincubated with 100 μg/mL RNPs for 6 h (Pre RNPs), followed by medium washout and stimulation with M5 cytokines for 48 h. The cell number was measured via CCK-8 assay. Data are represented as the mean ± SD (n = 3), $^{**}p < 0.01$ and $^{***}p < 0.001$ vs. phosphate-buffered saline (PBS).

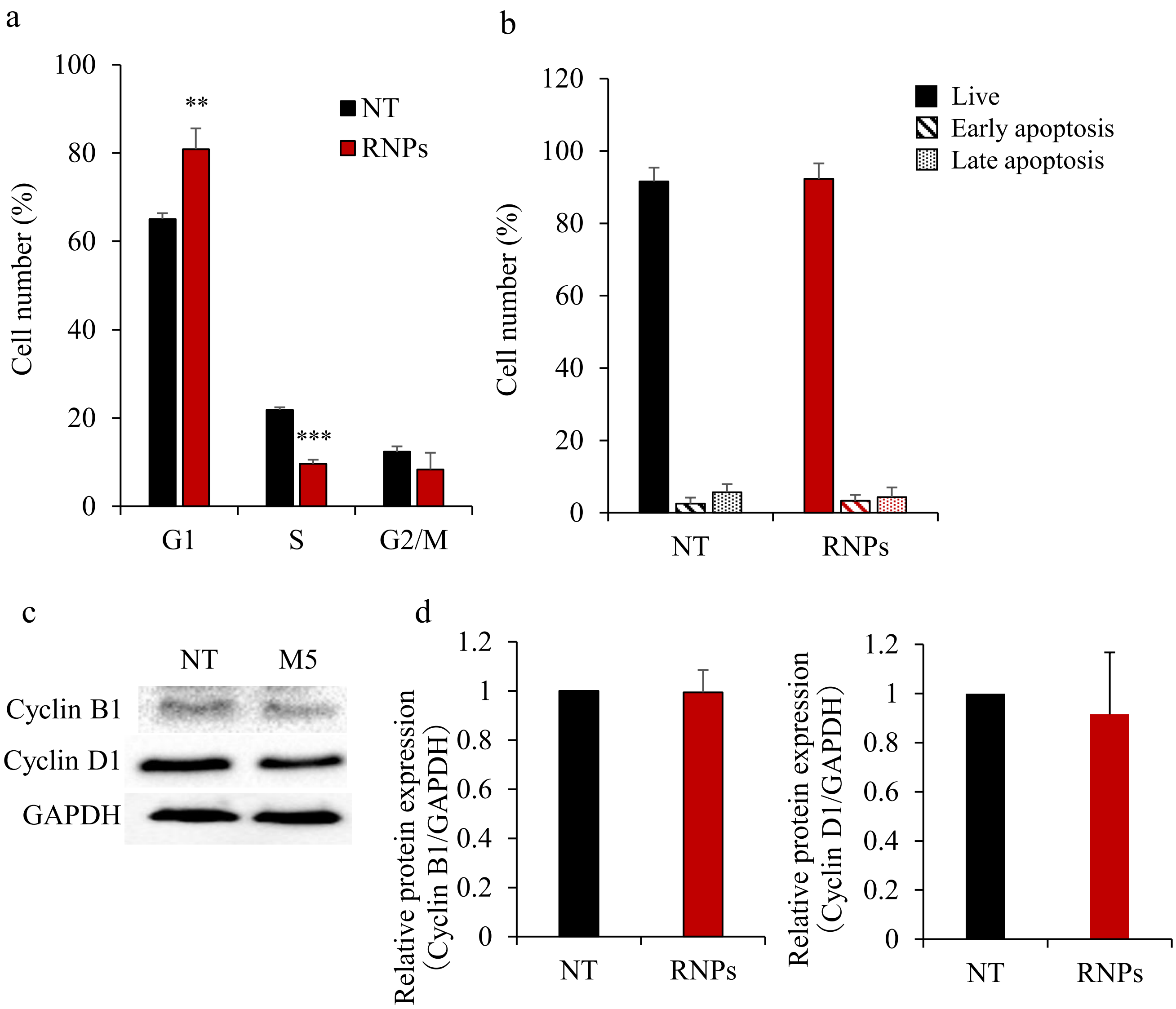

Figure S5. (a) Cell cycle distribution of RNP-treated HaCaT cells. After 48-h treatment, the cells were stained with the Cell Cycle Assay Solution Blue and analyzed via flow cytometry using the FlowJo software. Data are represented as the mean ± SD (n = 3). $^{**}p < 0.01$ and $^{***}p < 0.001$ vs. NT. NT, no treatment. (b) Apoptosis assay of RNP-treated HaCaT cells. After 48-h treatment, the cells were analyzed using the apoptosis detection kit. Percentages of live, early apoptotic, and late apoptotic cells were determined using the FlowJo software. Data are represented as the mean ± SD ($n = 3$). (c) Western blotting analysis of cyclin B1, cyclin D1, and glyceraldehyde-3-phosphate dehydrogenase (GAPDH) levels in HaCaT cells treated with 50 µg/mL of RNPs for 48 h. (d) Band intensities of cyclin B1 and D1 determined using the ImageJ software and normalized to that of GAPDH. Data are represented as the mean ± SD ($n = 3$).

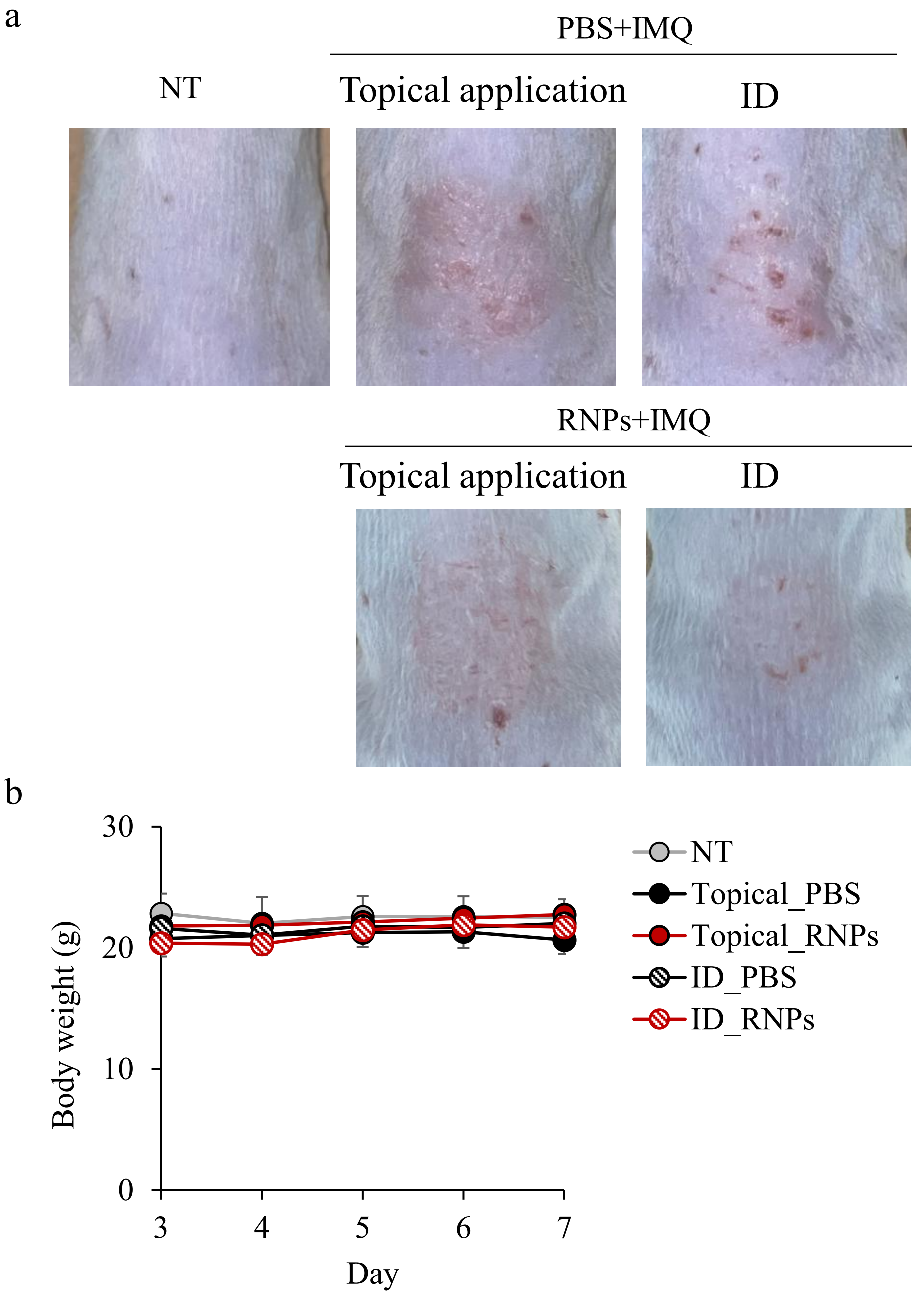

Figure S6. (a) Representative images of dorsal skin lesions after 7-day treatment. IMQ-induced psoriatic mice were treated with RNPs via topical application or intradermal (ID) injection using hollow microneedles. Mice treated with PBS served as negative controls. (b) Body weights of psoriatic skin model mice during the treatment period. no treatment (NT), PBS + IMQ applied topically (topical_PBS), PBS + IMQ via intradermal injection (ID_PBS), RNPs + IMQ applied topically (topical_RNPs), and RNPs + IMQ via intradermal injection (ID_RNPs). Data are represented as the mean ± SD (n = 4).

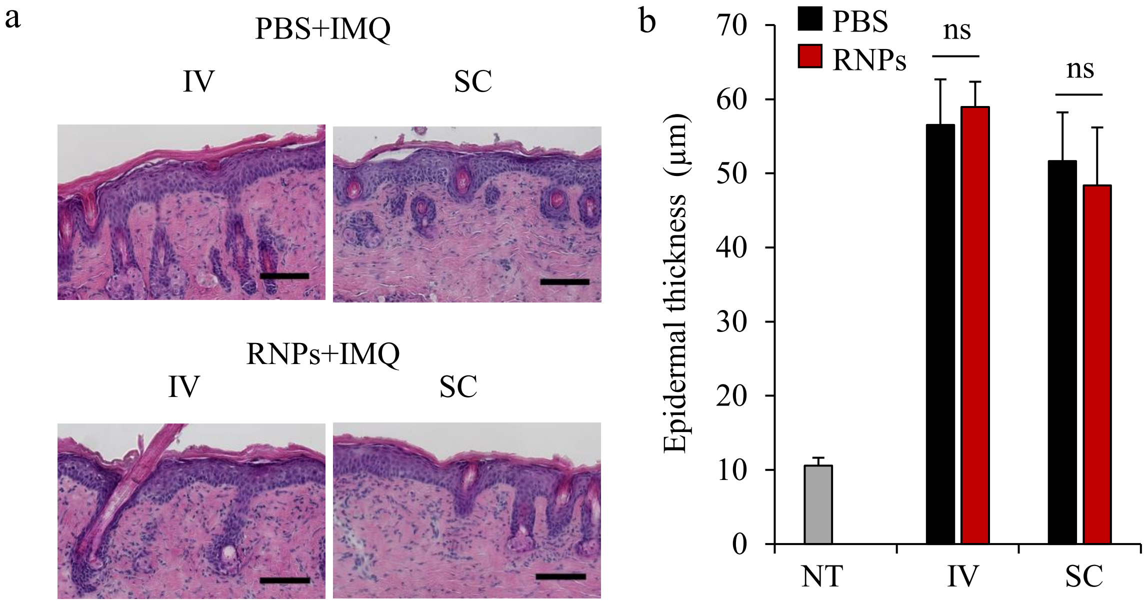

Figure S7. (a) Histological analysis of RNP-treated psoriatic skin in IMQ-induced psoriasis model mice. RNPs were administered via intravenous (IV) and subcutaneous (SC) injection (100 μg/mL) for seven consecutive days. Skin samples were stained with hematoxylin and eosin (H&E). Scale bars, 100 μm. (b) Quantification of epidermal thickness in RNP-treated psoriatic skin using the BZ-X Analyzer imaging software. Data are represented as the mean ± SD (n = 4). *$p < 0.05$; ns, not significant.

Supplementary Table S1. The Sequences of siRNAs.

siRNA	Target Gene	Sequence
siDNM2	Dynamin 2	5′-CCAACAUGGACCUGGCCAAtt-3′<br>5′-UUGGCCAGGUCCAUGUUGGtt-3′
siRac1	Ras-related C3 botulinum toxin substrate 1	5′-GGAACUAAACUUGAUCUUAGGGAtg-3′<br>5′-CAUCCCUAAGAUCAAGUUUAGUUCCCA-3′

Lowercase letters (tt, tg) indicate deoxynucleotide overhangs at the 3′ end of each strand.

Supplementary Table S2. The primer sequence for RT-qPCR.

Gene	Primer (5`→3`)
Human	
KRT6_F	GGGTTTCAGTGCCAACTCAG
KRT6_R	CCAGGCCATACAGACTGCGG
IFN-γ_F	ACGCTTGAAGACCTGGCTGA
IFN-γ_R	TTTGACAGTGCTGCTTGTGGA
IL-1β_F	TGATGGCTTATTACAGTGGCA
IL-1β_R	TGGTCGGAGATTCGTAGCTG
GAPDH_F	GGCACAGTCAAGGCTGAGAATG
GAPDH_R	ATGGTGGTGAAGACGCCAGTA
Mouse	
TNF-α_F	GCCTCTTCTCATTCCTGCTT
TNF-α_R	TGATGAGAGGGAGGCCATTTG
IL-1β_F	CCAGGATGAGGACATGAGCAC
IL-1β_R	TGTTGTTCATCTCGGAGCCTGTA
GAPDH_F	TGTGTCCGTCGTGGATCTGA
GAPDH_R	TTGCTGTTGAAGTCGCAGGAG
